# Estimating carrier rates and prevalence of porphyria-associated gene variants in the Chinese population based on genetic databases

**DOI:** 10.1186/s13023-024-03287-7

**Published:** 2024-09-12

**Authors:** Yinan Wang, Nuoya Li, Songyun Zhang

**Affiliations:** 1 Department of Basic Medicine, Hebei Medical University, 361 Zhongshan East Road, Chang’an District, Shijiazhuang, 050011 Hebei Province China; 2 Department of Public Health, Hebei Medical University, 361 Zhongshan East Road, Chang’an District, Shijiazhuang, 050011 Hebei Province China; 3 Hebei Key Laboratory of Rare Diseases, Shijiazhuang, 050000 Hebei China; 4 Porphyria Multi Disciplinary Team of the Second Hospital of Hebei Medical University, Shijiazhuang, 050000 Hebei China

**Keywords:** Porphyria, ChinaMAP, Genetics, Prevalence rate, Carrier rate

## Abstract

Porphyria is a group of rare metabolic disorders caused by mutations in the genes encoding crucial enzymes in the heme biosynthetic pathway. However, the lack of comprehensive genetic analysis of porphyria patients in the Chinese population makes identifying and diagnosing carriers of the condition challenging. Using the ChinaMAP database, we determined the frequencies of P/LP porphyria-associated gene variants according to the ACMG guidelines. We also calculated the carrier rates and prevalence of each type of porphyria in the Chinese population under Hardy–Weinberg equilibrium. Compared with the variants in the gnomAD database, the genetic spectrum of porphyria-related P/LP variants in the Chinese population is distinct. In the ChinaMAP database, we identified 23 variants. We estimated the carrier rates for autosomal dominant porphyrias (AIP, HCP, VP, PCT) in the Chinese population to be 1/1059, 1/1513, 1/10588, and 1/1765, respectively. For autosomal recessive porphyrias (ADP, EPP, HEP, CEP), the estimated carrier rates were 1/5294, 1/2117, 1/1765, and 1/2647, respectively, with predicted prevalence rates of 8.92 × 10^−9^, 7.51 × 10^−5^, 8.02 × 10^−8^, and 3.57 × 10^−8^, respectively. Notably, 12 of the variants we identified were unique to the Chinese population. The predicted prevalence rate of EPP was the highest among the various types of porphyria in the Chinese population, while the others were moderate to low. This is the first comprehensive genetic study on porphyria in the Chinese population. Clarifying the genetic characteristics of various porphyria types among the Chinese population provides scientifically sound reference data for both research and genetic screening to identify porphyria carriers.

## Introduction

Porphyria is a collection of rare metabolic disorders resulting from mutations in genes that control enzymes affecting the heme biosynthesis pathway [1]. These disorders are typically inherited in an autosomal dominant (AD), autosomal recessive (AR), or X-linked manner.

**Fig. 1:**
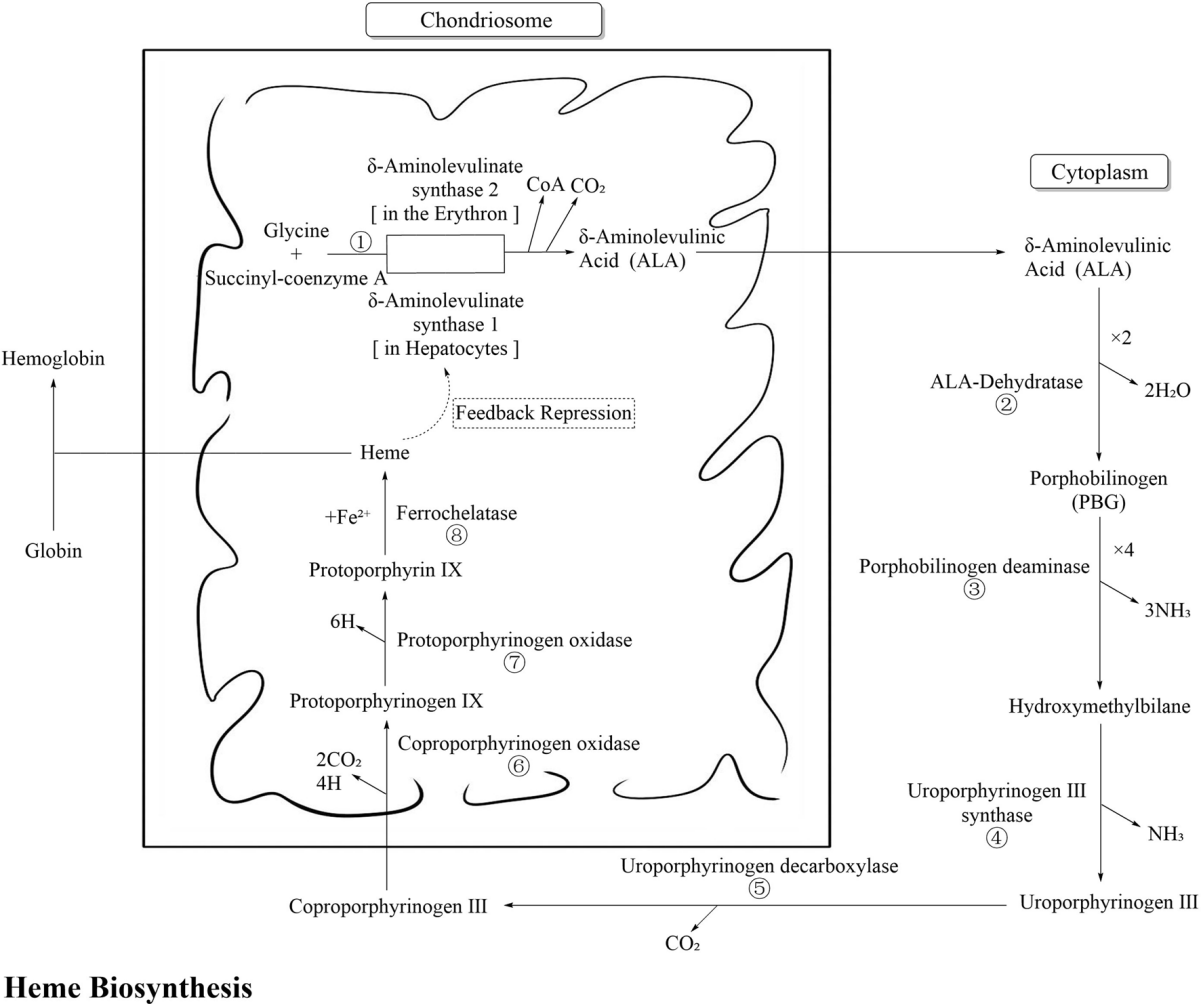
Biosynthesis Process of Heme. (Source from: < Biochemistry and Molecular Biology >, Zhou Chunyan, Yao Libo (ED.), 9 Ed, Beijing: People's Medical Publishing House, 2018). (*Note* Enzyme ①–⑧ are coded by *ALAS2*, *ALAD*, *HMBS*, *UROS*, *UROD*, *CPOX*, *PPOX* and. *FECH* genes respectively.)

The biosynthesis process of heme is shown in Fig. 1 and detailed below. The enzyme in step ① is coded by *ALAS1* and *ALAS2*. *ALAS1* express in the liver and undergoes negative-feedback regulation depending on the cellular heme concentration, which is particularly relevant to acute hepatic porphyrias (AHPs). *ALAS2* is an erythroid-specific gene, and mutations in this gene may cause X-linked protoporphyria (XLP) [2]. In this article, we mainly discuss the effect of *ALAS2* mutations on the prevalence of XLP.

The enzymes in steps ③, ⑥, and ⑦ are coded by *HMBS*, *CPOX*, and *PPOX*, respectively. Mutations in these genes may cause acute intermittent porphyria (AIP), hereditary coproporphyria (HCP) and variegate porphyria (VP), respectively, which exhibit AD inheritance.

The enzymes in steps ②, ④, and ⑧ are coded by *ALAD*, *UROS,* and *FECH,* respectively. Mutations in these genes may cause δ-aminolaevulinic acid dehydratase porphyria (ADP), congenital erythropoietic porphyria (CEP), and erythropoietic protoporphyria (EPP), respectively, which exhibit AR inheritance. Notably, EPP has a unique pathogenesis. It can result from a homozygous mutation, but more than 95% of EPP patients are compound heterozygous for a pathogenic mutation and the *FECH* low-expression single-nucleotide polymorphism (SNP) locus c.315-48T>C [2]

The enzyme in step ⑤ is encoded by *UROD*. Heterozygous *UROD* variants may cause AD porphyria cutanea tarda (PCT), and compound heterozygous *UROD* variants may cause AR hepatoerythropoietic porphyria (HEP) [3]. In the majority PCT patients, no genetic defects are present. Only approximately 20% of patients have a mutation in one of the alleles of the *UROD* gene, which can cause a reduction in the activity of enzyme ⑤ to less than 20% [4]. PCT is also considered an iron-related disorder. The disease becomes active when patients are exposed to predisposing factors that cause hepatic iron overload, including excess alcohol consumption, oestrogen use, infections (HCV, HIV, etc.), and smoking. It has been postulated that the hepatic activity of UROD is markedly reduced during active disease due to the formation of uroporphomethene, an iron-oxidized product of uroporphyrinogen, which acts as a reversible inhibitor of UROD activity [2].

Because of genetic heterogeneity, the carrier rates and prevalence rates of different types of porphyria vary among racial groups, making assessment complex. Information on the genetics of porphyria has primarily come from a handful of European countries, including France, Finland and Sweden, as well as from the Japanese population in Asia. The data from these regions have been largely limited to case reports and small series of studies. In the available studies, the prevalence of porphyria has mainly been estimated from epidemiological surveys and patient registries, with only a few studies based on genetic databases. The prevalence of inherited rare diseases may be difficult to accurately estimate using traditional methods [5]; specifically, late-onset and slow-progressing forms of rare diseases may be underestimated. Genetic studies of porphyria in Chinese populations are also limited.

The present study utilized the China Metabolic Analysis Project (ChinaMAP) biobank as a genetic data source for the normal Chinese population, and the allele frequencies (AFs) of pathogenic (P)/likely pathogenic (LP) variants were interpreted and screened according to the American College of Medical Genetics and Genomics (ACMG) guidelines. The carrier rates and prevalence rates of each type of porphyria in the Chinese population were predicted using the Hardy‒Weinberg equilibrium (HWE). Moreover, the genetic characteristics of each type of porphyria in the Chinese population were determined by comparing the results with those of eight other ethnicities in the Genome Aggregation Database (gnomAD) Genome V3.0.

This study aimed to conduct the first comprehensive genetic study of porphyria in the Chinese population utilizing the ChinaMAP genetic database. By comparison with various ethnic groups in gnomAD, we aimed to illustrate the genetic characteristics of various types of porphyria in the Chinese population. These results will provide scientific and reliable reference data for clinical research and genetic screening of porphyria carriers.

## Methods

### Screening and interpretation of P/LP gene variants in different types of porphyria

ChinaMAP (www.mbiobank.com) is a biobank of the Chinese population based on a China-wide cohort study of metabolic phenotypic data from various regions and ethnic groups. The analysis of in-depth whole-genome sequencing (WGS) data from 10,588 participants, which includes 21,176 alleles, was completed in this project. The ChinaMAP database is a valuable tool for researching and pinpointing potential pathogenic mutations that cause diseases. Its goal is to identify both common and rare but impactful mutations in Chinese populations, particularly in unknown genes and metabolic pathways related to metabolic diseases and their complications. This information could help identify new diagnostic and treatment approaches for patients who are at high risk of specific non-infectious chronic diseases.

The genetic data for the normal Chinese population were sourced from ChinaMAP (accessed on 19 April 2021), while genetic data for other ethnic populations, including East Asian (EAS), Ashkenazi Jewish (ASJ), Mixed American (AMR), African/African American (AFR), Amish (AMI), Finnish (FIN), non-Finnish European (NFE), South Asian (SAS), and other (OTH), were obtained from the gnomAD Genome V3.0 database. The data in both biobanks are derived from population-based studies utilizing WGS data. P/LP variants in both databases were interpreted and screened according to the ACMG guidelines, ensuring the comparability of the data.

The gene variant nomenclature followed the Human Genome Variation Society (HGVS) standards, specifically GRCh38/hg38. DNA and protein sequence numbering was carried out independently based on the reference sequence.

The process of screening and interpreting porphyria-associated genetic variants generally involves the following steps:

1. Using the rating results given by InterVar software and the annotation information of the ClinVar database and Human Gene Mutation Database (HGMD) as references, we manually screened for genetic variants associated with porphyria in exonic and splice regions with a small AF (≤ 0.05). We used databases such as ChinaMAP, the 1000 Genomes Project, the Exome Aggregation Consortium, the Exome Variant Server (EVS), and gnomAD Genome V3.0. We utilized various computer tools, including SIFT, PolyPhen2_HDIV, PolyPhen2_HVAR, LRT, MutationTaster, and MutationAssessor, to predict the pathogenicity of the screened variants. These tools were used to determine whether a mutation disrupts the structure and function of a protein or affects splicing.
2. In this study, we estimated the Rare Exome Variant Ensemble Learner (REVEL) and s-PP3 scores for each mutation. REVEL is a method that predicts rare missense mutations by combining the results of multiple software programs to generate a score between 0 and 1. A higher REVEL score indicates a greater likelihood that the variant is responsible for the disease [6]. In this study, we used a REVEL score > 0.7 as the threshold for applying the ACMG's PP3 criterion. Additionally, we developed the s-PP3, a unique scoring system that helps interpret ratings. s-PP3 is a composite score based on the predictions of five splicing prediction software programs (dbscSNV_ADA, dbscSNV_RF, MMSplice, MaxEnt, SpliceAI), with 1 point awarded for each software package that predicts the effect of the mutation on splicing.
3. After completing these steps, the variants were classified as either P, LP or a variant of uncertain significance (VUS-P) based on the ACMG guidelines and the suggestions of the Sequence Variant Interpretation (SVI) Working Group of the Clinical Genome Resource (ClinGen).

### Prediction of the carrier rate and prevalence of pathogenic gene variants of each type of porphyria

Variants classified as P/LP according to the ACMG guidelines have corresponding AFs. By applying the HWE equation (p^2^ + 2pq + q^2^ = 1), the carrier rate and prevalence rate for each porphyria-associated gene variant can be calculated. Assuming that q represents the AF of the P/LP variant, the carrier rate of the pathogenic variant responsible for AD porphyria can be estimated as 2pq (with p approximated to be 1), and the prevalence rate is calculated as the product of the carrier rate and the epistasis rate.

The prevalence of homozygosity for the pathogenic variant responsible for AR porphyria is determined by squaring the frequency of the pathogenic variant, denoted as q^2^. Additionally, the prevalence of compound heterozygosity is calculated as the square of the sum of the AFs of the pathogenic variant genes minus the sum of the squares of the AFs of the pathogenic variants: ${\left(\sum_{i=1}^{n}{q}_{i}\right)}^{2}-\sum_{\text{i}=1}^{\text{n}}{\left({\text{q}}^{2}\right)}_{\text{i}}$.

AD porphyrias are exceptionally rare, typically presenting with early onset and severe symptoms, and have been reported on a case-by-case basis. Therefore, individuals with AD porphyria were not included in the prevalence calculations for this study. EPP is an AR porphyria with a distinct pathogenesis, stemming from either a homozygous state or a compound heterozygous state involving a pathogenic mutation and the *FECH* low-expression SNP locus c.315-48T>C. The frequency of the low-expression SNP locus c.315-48T>C varies across populations, thereby influencing the prevalence of EPP in different populations to some extent.

In this study, we calculated the prevalence of homozygosity or compound heterozygosity for pathogenic *FECH* variants, as well as the prevalence of compound heterozygosity for pathogenic *FECH* variants and the low-expressing SNP locus c.315-48T>C. These two results were combined to determine the total prevalence of EPP.

Because of the limited sample size, we utilized SPSS software for data analysis and the Clopper–Pearson Exact method to determine the 95% confidence intervals (95% CIs) to guarantee the reliability of the predictions.

### Comparative analysis of the distribution of porphyria-associated genetic variants in the Chinese population

In this study, the distribution of P/LP variants in porphyria-related genes and the predicted carrier and prevalence rates of each type of porphyria in the Chinese population were analysed. These rates were then compared with those of other populations, including EAS, ASJ, AMR, AFR, AMI, FIN, NFE, SAS, and OTH populations. The genetic characteristics of porphyria in the Chinese population, including specific sites and predicted carrier/prevalence levels, among others, were highlighted in a comparison of data from nine different ethnic groups.

## Results

### Overall porphyria-related gene variants in ChinaMAP

**Fig. 2:**
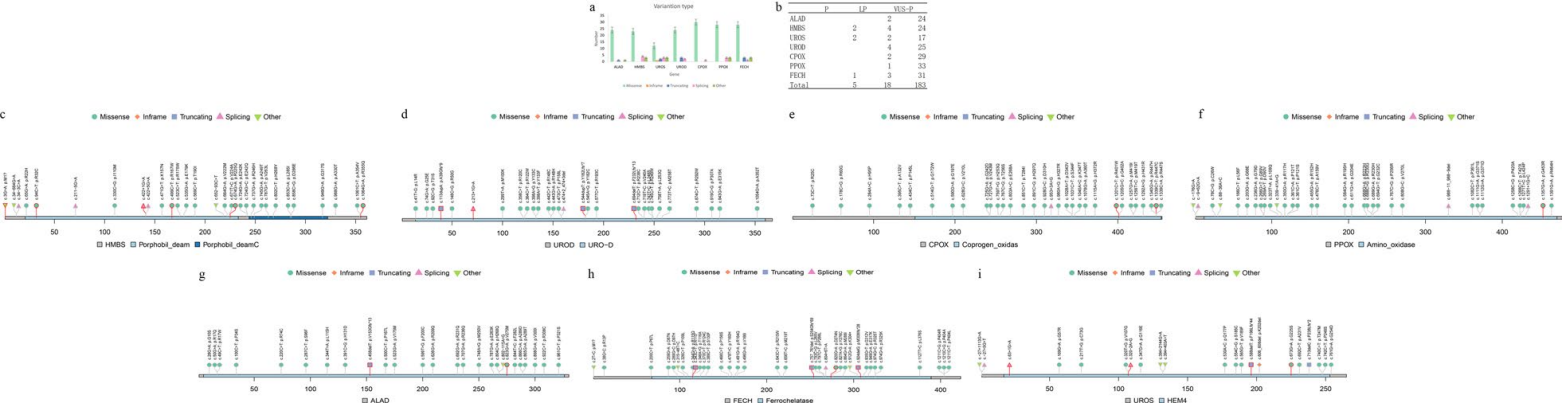
Information of P/LP and VUS-P porphyria-associated gene variants in the ChinaMAP database. (*Note* **a**, **b** Variant types and distribution of P/LP and VUS-P porphyria-associated variants in different genes in the ChinaMAP database. The error bars represent the mean plus or minus the standard error. **c**–**i** Protein amino acid map of P/LP + VUS-P variations of *HMBS*, *UROD*, *CPOX*, *PPOX, ALAD*, *UROS*, and *FECH*. The horizontal axis represents the protein amino acid position, the red frame represents P/LP variations, and different legends represent different types of variations. Protein data was from UniProt and Pfam.)

In ChinaMAP, eight porphyria-associated genes were examined, resulting in the identification of 206 P, LP, and VUS-P variants based on the ACMG guidelines. Among these variants, there were 5 P variants, 18 LP variants, and 183 VUS-P variants. The most common type of mutation was missense mutations, with 169 variants. In addition to missense mutations, there were 14 splice mutations, 9 truncation mutations, 1 in-frame insertion/deletion, and 13 other types of variants. The distribution of each type of variant in every gene is shown in Fig. 2a, while Fig. 2b depicts the distributions of the P/LP and VUS-P variants in each gene.

### AD Porphyria-associated genetic variants

#### Characteristics of P/LP variants in AD-inherited genes in ChinaMAP

A total of 13 P/LP AD-inherited variants related to porphyria were screened in ChinaMAP, with 6 for *HMBS*, 4 for *UROD*, 2 for *CPOX*, and 1 for *PPOX*. The greatest number of variants was detected in *HMBS*, while the lowest was detected in *PPOX*. The carrier rates of pathogenic variants for each type of AD porphyria in the Chinese population were as follows: AIP, 1/1059 (9.445 × 10^−4^, 4.527 × 10^−4^–1.735 × 10^−3^); PCT, 1/1765 (5.664 × 10^−4^, 2.079 × 10^−4^–1.233 × 10^−3^); HCP, 1/1513 (6.611 × 10^−4^, 2.657 × 10^−4^–1.361 × 10^−3^); and VP, 1/10588 (9.44 × 10^−5^, 2.4 × 10^−6^–5.261 × 10^−4^). AIP had the highest predicted carrier rate, while VP had the lowest. The most prevalent AD-inherited variant was c.1339C>T (p.Arg447Cys) in *CPOX*, with an allele frequency of 0.0002833 in the normal Chinese population. Missense mutations were the most common type of variant in these loci. Table 1 shows the information for all AD porphyria-associated P/LP variant loci in ChinaMAP, and the details of the P/LP and VUS-P variants for each gene are presented in Fig. 2c–f.

**Table 1:** Information of all porphyria-associated P/LP variant loci in ChinaMAP

ACMG evidence of pathogenicity	REVEL	s-PP3	ChinaMap_Num	ChinaMap_Frq	Predict carrying rate of P/LP variants in CHI	Homozygous prevalence	Compound heterozygous prevalence	Total predict carrying rate of P/LP variation	Total predict prevalence of P/LP variation
PVS1_Moderate, PM2_supporting, PS3_Moderate:Chen et al. found that this variant affects the length of the protein [PMID: 7962538]; PS4_supporting:The literature reports the detection of this variant in several patients [PMID: 27539938, 7962538]	0.696	0.5 [spliceAI]	1	0.0000472	0.0000944 (1/10588)	–	–	0.0009444 (0.0004527–0.0017353) (1/1059)	–
PM2_supporting, PP3; PM5; PM1	0.852	–	1	0.0000472	0.0000944 (1/10588)	–	–		
PVS1, PM2_supporting		3 [ADA, RF, spliceAI]	2	0.0000944	0.0001888 (1/5294)	–	–		
PM2_supporting, PP3, PS3	0.851	1 [maxent]	4	0.0001889	0.0003777 (1/2647)	–	–		
PM2_supporting, PP3; PM5; PS3: Experiments have shown that the variant affects enzyme activity [PMID: 23815679; 15534187]; PS4_Moderate:Multiple reports in the literature have detected the variant [PMID: 1496994, 23815679]	0.943	–	1	0.0000472	0.0000944 (1/10588)	–	–		
PM2_supporting, PP3; PM1; PM5; PS3: Experiments have shown that the variant affects enzyme activity [PMID: 29360981]; PS4_supporting:The literature reports detection of this variant in 4 patients [PMID: 12372055; 29360981]	0.915	–	1	0.0000472	0.0000944 (1/10588)	–	–		
PVS1, PM2_supporting		–	2	0.0000944	0.0001888 (1/5294)	–	–	0.0005664 (0.0002079–0.0012327) (1/1765)	–
PVS1, PM2_supporting		3 [ADA, RF, spliceAI]	1	0.0000472	0.0000944 (1/10588)	–	–		
PVS1, PM2_supporting		–	1	0.0000472	0.0000944 (1/10588)	–	–		
PVS1, PM2_supporting		–	2	0.0000944	0.0001888 (1/5294)	–	–		
PM2_supporting, PP3, PS3: This variant reduces enzyme activity [PMID: 11309681]	0.856	–	6	0.0002833	0.0005666 (3/5294)	–	–	0.0006610 (0.0002657–0.0013612) (1/1513)	–
PM2_supporting, PP3, PS3: This variant reduces enzyme activity [PMID: 11309681, 24078084]	0.948	1 [spliceAI]	1	0.0000472	0.0000944 (1/10588)	–	–		
PM2_supporting, PP3, PS3: This variant reduces enzyme activity [PMID: 11929051, 21048046]	0.988	–	1	0.0000472	0.0000944 (1/10588)	–	–	0.0000944 (0.0000024–0.0005261) (1/10588)	–
PM2_supporting, PP3, PS3: This variant reduces enzyme activity [PMID: 11342419]; PM3: Conpound heterozygous variation with G133R was detected [PMID: 2063868]	0.832	–	1	0.0000472	0.0000944 (1/10588)	0.00000000222784 (1/448422976)	0.00000000445568 (1/224211488)	0.0001888 (0.0000229–0.0006821) (1/5294)	0.00000000891136 (0.00000000247184–0.00000002276626) (1/112105744)
PVS1, PM2_supporting			1	0.0000472	0.0000944 (1/10588)	0.00000000222784 (1/448422976)			
PVS1, PM2_supporting		–	1	0.0000472	0.0000944 (1/10588)	0.00000000222784 (1/448422976)	0.00000002673408 (3/112105744)	0.0003776 (0.0001029–0.0009668) (1/2647)	0.00000003564544 (0.00000002037835–0.00000005790729) (1/28026436)
PVS1, PM2_supporting		3 [ADA, RF, spliceAI]	1	0.0000472	0.0000944 (1/10588)	0.00000000222784 (1/448422976)			
PM2_supporting, PP3, PS3: The variant affects enzyme activity [PMID: 7860775]; PM3_strong:This variant was detected to be compound heterozygous with other variants [PMID: 7860775, 11254675, 19099412]	0.954	–	1	0.0000472	0.0000944 (1/10588)	0.00000000222784 (1/448422976)			
PVS1, PM2_supporting, PM3: This variant was detected to be compound heterozygous with other variants [PMID: 7860775]		5 [ADA, RF, ssplice, maxent, spliceAI]	1	0.0000472	0.0000944 (1/10588)	0.00000000222784 (1/448422976)			
PVS1, PM2_supporting		–	2	0.0000944	0.0001888 (1/5294)	0.00000000891136 (1/112105744)	0.00000005792384 (13/224211488)	0.0005664 (0.0002079–0.0012327) (1/1765)	0.00000008020224 (0.00000005624382–0.00000011122176) (1/12456194)
PVS1, PM2_supporting			1	0.0000472	0.0000944 (1/10588)	0.00000000222784 (1/448422976)			
PVS1, PM2_supporting			1	0.0000472	0.0000944 (1/10588)	0.00000000222784 (1/448422976)			
PVS1, PM2_supporting			2	0.0000944	0.0001888 (1/5294)	0.00000000891136 (1/112105744)			
PVS1, PM2_supporting		–	2	0.0000944	0.0001889 (1/5294)	0.00000000891136 (1/112105744)	0.00000004014069 (9/224211488)	0.0004722 (0.0001533–0.0011014) (1/2117)	0.00007511868016 (0.00000190173394–0.00041843963362) (1/13312)
PM2_supporting; PM3: Compound heterozygosity with multiple loci [PMID: 23364466, 16385445, 18787536]; PS3: The mutation reduces enzyme activity to 0.8% compared to the wild type [PMID: 18787536]; PP3	0.901		1	0.0000472	0.0000944 (1/10588)	0.00000000222784 (1/448422976)			
PVS1, PM2_supporting			1	0.0000472	0.0000944 (1/10588)	0.00000000222784 (1/448422976)			
PVS1, PM2_supporting, PP1:The variant was detected in two families [PMID: 24912412]			1	0.0000472	0.0000944 (1/10588)	0.00000000222784 (1/448422976)			
PS3: The mutation disrupts mRNA splicing [PMID: 11753383]; BA1			6732	0.3179070	–	–	0.00007506292921 (0.00000190030653–0.00041812561438) (1/13322)	–	

### Distribution and characteristics of AD-inherited P/LP variants in different ethnic populations

**Fig. 3:**
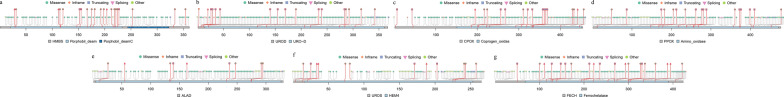
Protein amino acid map of P/LP + VUS-P variations of porphyria-associated gene variants in the gnomAD Genome V3.0 database. (*Note* Protein amino acid map of P/LP + VUS-P variations of *HMBS*, *UROD*, *CPOX*, *PPOX*, *ALAD*, *UROS*, and *FECH*. The horizontal axis represents the protein amino acid position, the red frame represents P/LP variations, and different legends represent different types of variations. Protein data was from UniProt and Pfam.)

In accordance with the ACMG guidelines, a total of 73 AD porphyria-associated P/LP variants were screened in gnomAD Genome V3.0. Specifically, 21 variants were screened in *HMBS*, 17 in *UROD*, 18 in *CPOX*, and 17 in *PPOX*. Notably, the greatest number of variants was found in *HMBS*, consistent with the results from ChinaMAP. The carrier rates of pathogenic mutations for AIP, PCT, HCP, and VP differed between ChinaMAP and gnomAD. The carrier rate for AIP was 1/1059 in ChinaMAP and 1/814 in gnomAD. For PCT, the carrier rate was 1/1765 in ChinaMAP and 1/3087 in gnomAD. The carrier rate of HCP was 1/1513 in ChinaMAP and 1/1023 in gnomAD. The carrier rate for VP was 1/10588 in ChinaMAP and 1/2985 in gnomAD. The most common AD-inherited variant in gnomAD was the c.1339C>T (p.Arg447Cys) variant in *CPOX*, which was also found in ChinaMAP. Similarly, the predominant type of variation observed at these loci was missense mutations. Table 2 contains all the relevant information for AD-inherited porphyria-associated P/LP variant loci in gnomAD Genome V3.0, and the subsequent figures (Fig. 3a–d) provide details on P/LP and VUS-P variants in each gene.

**Table 2:** Information of all porphyria-associated P/LP variant loci in gnomAD

Inheritance Mode	Gene (Type of Porphiria)	DNA change	AA change	ACMG Variant Assessment	Variant type	ACMG Evidence of Pathogenicity	HGMD_tag	REVEL	s-PP3	gnomAD Genome V3.0_Frq	Total predict carrying rate of P/LP variation	Total predict prevalence of P/LP variation
AD	HMBS (AIP)	c.1064G>A	p.Arg355Gln	LP	Nonsynonymous SNV	PM2_supporting, PP3, PS3	FP	0.85	1 [maxent]	0.0000628	0.0012283(0.0009846–0.0015121) (1/814)	–
		c.91G>A	p.Ala31Thr	LP	Nonsynonymous SNV	PM2_supporting, PM5, PP3, PM1, PS4_supporting:The variant was detected in several patients [PMID: 827054]	DM	0.99	–	0.0000070		
		c.345–1G>A	–	LP	Canonical splicing	PVS1, PM2_supporting	DM		5 [ADA, RF, ssplice, maxent, spliceAI]	0.0000070		
		c.347G>A	p.Arg116Gln	LP	Nonsynonymous SNV	PM2_supporting, PM5, PP3, PM1	DM	0.95	1 [spliceAI]	0.0000140		
		c.457C>T	p.Gln153*	LP	Stopgain	PVS1, PM2_supporting			0.5 [spliceAI]	0.0000070		
		c.532G>A	p.Asp178Asn	LP	Nonsynonymous SNV	PM2_supporting, PP3; PM5, PM1; PS4_supporting [PMID: 12372055, 9199558]	DM	0.86	–	0.0000279		
		c.655G>T	p.Asp178Asn	LP	Nonsynonymous SNV	PM2_supporting, PP3, PM1, PM5		0.87	–	0.0000419		
		c.754G>A	p.Ala252Thr	LP	Nonsynonymous SNV	PM2_supporting, PP3; PM5, PM1	DM	0.79	–	0.0000140		
		c.992C>T	p.Ala331Val	LP	Nonsynonymous SNV	PM2_supporting, PP3, PM1, PS3	DM	0.96	–	0.0000419		
		c.661G>A	p.Gly221Ser	LP	Nonsynonymous SNV	PM2_supporting, PP3; PM5; PM1		0.8	–	0.0000070		
		c.87+5G>A	–	LP	Splicing	PM2_supporting, PP3; PS3	DM		5 [ADA, RF, ssplice, maxent, spliceAI]	0.0000070		
		c.437_443del	p.Ser147Glufs*106	LP	Frameshift deletion	PVS1, PM2_supporting	DM		–	0.0000070		
		c.647G>C	p.Gly216Ala	LP	Nonsynonymous SNV	PM2_supporting, PP3; PM1; PM5		0.87	0.5 [spliceAI]	0.0000140		
		c.674G>A	p.Arg225Gln	P	Nonsynonymous SNV	PM2_supporting, PP3; PM1; PM5; PS3: Experiments have shown that the variant affects enzyme activity [PMID: 29360981]; PS4_supporting:The literature reports detection of this variant in 4 patients [PMID: 12372055; 29360981]	DM	0.92	–	0.0002234		
		c.346C>T	p.Arg116Trp	P	Nonsynonymous SNV	PM2_supporting, PM5, PP3, PM1, PS4	DM	0.95	1 [maxent]	0.0000070		
		c.500G>A	p.Arg167Gln	P	Nonsynonymous SNV	PM2_supporting, PP3; PM5, PS3; PS4_Moderate: [PMID: 15003823]	DM	0.93	3 [ADA, RF, spliceAI]	0.0000628		
		c.517C>T	p.Arg173Trp	P	Nonsynonymous SNV	PM2_supporting, PP3; PM5, PM1; PS4 [PMID: 15003823; 23815679]	DM	0.9	–	0.0000070		
		c.583C>T	p.Arg195Cys	P	Nonsynonymous SNV	PM2_supporting, PP3; PM5, PM1; PS3	DM	0.9	–	0.0000070		
		c.601C>T	p.Arg201Trp	P	Nonsynonymous SNV	PM2_supporting, PP3; PM5, PM1; PS3; PS4_Moderate [PMID: 12372055]	DM	0.93	1 [maxent]	0.0000349		
		c.613–1G>T	–	P	Canonical splicing	PVS1, PM2_supporting; PP1_Moderate: [PMID: 11030413]	DM		5 [ADA, RF, ssplice, maxent, spliceAI]	0.0000070		
		c.673C>T	p.Arg225*	P	Stopgain	PVS1, PM2_supporting; PS3, PS4_supporting	DM		1.5 [maxent, spliceAI]	0.0000070		
	UROD (PCT)	c.346C>T	p.Gln116*	LP	Stopgain	PVS1, PM2_supporting	DM	0.02	–	0.0000070	0.0003239(0.0002039–0.0004827) (1/3087)	–
		c.842G>A	p.Gly281Glu	LP	Nonsynonymous SNV	PM2_supporting, PP3, PS3: The mutation reduced the enzyme activity, which was significantly lower in homozygote compared to the wild type [PMID: 8644733]; PM3: Heterozygote of the mutation was detected	DM	0.98	–	0.0000070		
		c.912C>A	p.Asn304Lys	LP	Nonsynonymous SNV	PM2_supporting, PP3, PS3: The mutation reduced the enzyme activity to 19.5% compared to the wild type [PMID: 9792863]	DM	0.79	–	0.0000070		
		c.397_398del	p.Tyr133Cysfs*34	LP	Frameshift deletion	PVS1, PM2_supporting	DM		–	0.0000070		
		c.424C>T	p.Arg142*	LP	Stopgain	PVS1, PM2_supporting	DM	0.11	–	0.0000070		
		c.616C>T	p.Gln206*	LP	Stopgain	PVS1, PM2_supporting	DM		1 [spliceAI]	0.0000070		
		c.649dupT	p.Glu218*	LP	Stopgain	PVS1, PM2_supporting	DM		2 [maxent, spliceAI]	0.0000070		
		c.1007A>G	p.Asn336Ser	LP	Nonsynonymous SNV	PM2_supporting, PP3, PS3: The mutation reduced the enzyme activity to 36.89% compared to the wild type [PMID: 17627795]	DM	0.93	–	0.0000279		
		c.1A>G	p.Met1?	LP	Startloss	PM2_supporting, PVS1_moderate, PP1_strong:The mutation was isolated in the family, showing co–segregation [PMID: 18462440]	DM	0.66	–	0.0000070		
		c.21–2A>C	–	LP	Canonical splicing	PVS1, PM2_supporting			3 [ADA, RF, spliceAI]	0.0000072		
		c.23_24insTCAG	p.Gly10Serfs*9	LP	Frameshift insertion	PVS1, PM2_supporting			–	0.0000070		
		c.27delG	p.Gly10Valfs*5	LP	Frameshift deletion	PVS1, PM2_supporting			–	0.0000071		
		c.30_31insCACCAGCTGCTTCGCATCCTCACTGATGCTCTGGTCCCATATCTGGTAGGACAAGTGGTGGC	p.Phe11Hisfs*25	LP	Frameshift insertion	PVS1, PM2_supporting			–	0.0000082		
		c.62delG	p.Arg21Glnfs*48	LP	Frameshift deletion	PVS1, PM2_supporting			1.5 [maxent, spliceAI]	0.0000279		
		c.820C>T	p.Gln274*	LP	Stopgain	PVS1, PM2_supporting		0.04	–	0.0000070		
		c.850T>C	p.Trp284Arg	LP	Nonsynonymous SNV	PM2_supporting, PP3, PS3: The mutation reduced the enzyme activity to 24% compared to the wild type [PMID: 23545314]; PS4_supporting:The variant was detected in 4 patients [PMID: 19233912, 23545314]	DM	0.45	–	0.0000070		
		c.494T>G	p.Met165Arg	P	Nonsynonymous SNV	PM2_supporting, PP3, PS3: The mutation reduced the enzyme activity to 1.36% compared to the wild type [PMID: 9792863]	DM	0.97	–	0.0000070		
	CPOX (HCP)	c.1339C>T	p.Arg447Cys	LP	Nonsynonymous SNV	PM2_supporting, PP3, PS3: This variant reduces enzyme activity [PMID: 11309681]	DM	0.86	–	0.0002720	0.0009769(0.0007616–0.0012340) (1/1023)	–
		c.1201C>T	p.Arg401Trp	LP	Nonsynonymous SNV	PM2_supporting, PP3, PS3: This variant reduces enzyme activity [PMID: 11309681, 24078084]	DM	0.95	1 [spliceAI]	0.0000070		
		c.1277 + 3A>G	–	LP	Splicing	PM2_supporting, PP3; PS3: This mutation disrupts splicing [PMID: 9454777]; PM3: Conpound heterozygous variation with K404E was detected [PMID: 9454777]	DM		1 [spliceAI]	0.0000209		
		c.877G>A	p.Ala293Thr	LP	Nonsynonymous SNV	PM2_supporting, PP3, PS3: This variant reduces enzyme activity [PMID: 11309681];	DM	0.9	–	0.0000140		
		c.601G>A	p.Glu201Lys	LP	Nonsynonymous SNV	PM2_supporting, PP3, PS3: This variant reduces enzyme activity [PMID: 11309681];	DM	0.95	–	0.0000279		
		c.131_132insGCAGC	p.Gly45Glnfs*93	LP	Frameshift insertion	PVS1, PM2_supporting	DM		–	0.0000140		
		c.1006_1007insCC	p.Gly336Alafs*27	LP	Frameshift insertion	PVS1, PM2_supporting			–	0		
		c.1005_1006insC	p.Gly336Argfs*5	LP	Frameshift insertion	PVS1, PM2_supporting			1 [maxent]	0		
		c.997_1001del	p.Gly333Trpfs*6	LP	Frameshift deletion	PVS1, PM2_supporting			–	0		
		c.993delG	p.Arg332Glyfs*30	LP	Frameshift deletion	PVS1, PM2_supporting			–	0		
		c.953G>A	p.Trp318*	LP	Stopgain	PVS1, PM2_supporting			5 [ADA, RF, ssplice, maxent, spliceAI]	0.0000070		
		c.916C>T	p.Gln306*	LP	Stopgain	PVS1, PM2_supporting	DM		0.5 [spliceAI]	0.0000070		
		c.688A>T	p.Lys230*	LP	Stopgain	PVS1, PM2_supporting			–	0.0000070		
		c.663_667del	p.Met222Lysfs*9	LP	Frameshift deletion	PVS1, PM2_supporting			–	0.0000070		
		c.607G>A	p.Ala203Thr	LP	Nonsynonymous SNV	PM2_supporting, PP3, PS3: K404E [PMID: 11309681]	DM	0.95	1.5 [maxent, spliceAI]	0.0000070		
		c.557–2A>G	–	LP	Canonical splicing	PVS1, PM2_supporting			5 [ADA, RF, ssplice, maxent, spliceAI]	0.0000070		
		c.8delT	p.Leu3Cysfs*6	LP	Frameshift deletion	PVS1, PM2_supporting			–	0.0000070		
		c.1210A>G	p.Lys404Glu	P	Nonsynonymous SNV	PM2_supporting, PP3; PP1_Moderate:The variant was detected in a family of 3 patients, showing co–segregation [PMID: 16159891]; PM3_strong:Conpound heterozygous variation with c.1277 + 3A>G and many homozygotes were detected [PMID: 16159891; 6886003]; PS3: This variant reduces enzyme activity [PMID: 16159891]	DM	0.94	–	0.0000838		
	PPOX (VP)	c.1357G>A	p.Gly453Arg	LP	Nonsynonymous SNV	PM2_supporting, PP3, PS3: This variant reduces enzyme activity [PMID: 11929051, 21048046]	DM	0.99	–	0.0000070	0.0003350(0.0002147–0.0004986) (1/2985)	–
		c.35T>C	p.Ile12Thr	LP	Nonsynonymous SNV	PM2_supporting, PP3, PS3: This variant affects enzyme activity [PMID: 11286631]	DM	0.93	–	0.0000488		
		c.565C>T	p.Gln189*	LP	Stopgain	PVS1, PM2_supporting,	DM		–	0.0000070		
		c.649C>T	p.Arg217Cys	LP	Nonsynonymous SNV	PM2_supporting, PP3, PM5:At the same locus, the p.Arg168His variant is pathogenic; PS3: This variant reduces enzyme activity [PMID: 21048046]	DM	0.63	0.5 [spliceAI]	0.0000209		
		c.1072G>A	p.Gly358Arg	LP	Nonsynonymous SNV	PM2_supporting, PP3, PM5:At the same locus, the p.Arg168His variant is pathogenic; PS3: This variant reduces enzyme activity [PMID: 21048046, 9811936]	DM	0.48	0.5 [spliceAI]	0.0000070		
		c.532C>G	p.Leu178Val	LP	Nonsynonymous SNV	PM2_supporting, PP3, PS3: This variant reduces enzyme activity [PMID: 21048046]	DM	0.8	–	0.0000070		
		c.712C>T	p.Gln238*	LP	Stopgain	PVS1, PM2_supporting,			–	0.0000070		
		c.182dupT	p.Arg62*	LP	Stopgain	PVS1, PM2_supporting,			1 [spliceAI]	0.0000070		
		c.766_767insGG	p.Pro256Argfs*18	LP	Frameshift insertion	PVS1, PM2_supporting,			–	0.0000070		
		c.767_768insT	p.Val257Glyfs*24	LP	Frameshift insertion	PVS1, PM2_supporting,			1 [maxent]	0.0000070		
		c.1118G>A	p.Trp373*	LP	Stopgain	PVS1, PM2_supporting,		0.32	–	0.0000070		
		c.1123C>T	p.Gln375*	LP	Stopgain	PVS1, PM2_supporting	DM		–	0.0000070		
		c.1209_1210insATTTTTT	p.Lys404Ilefs*3	LP	Stopgain	PVS1, PM2_supporting,			–	0		
		c.1223delG	p.Ser408Thrfs*17	LP	Frameshift deletion	PVS1, PM2_supporting,			–	0.0000071		
		c.503G>A	p.Arg168His	P	Nonsynonymous SNV	PM2_supporting, PP3, PM5:At the same locus, the p.Arg168His variant is pathogenic; PS3: This variant reduces enzyme activity [PMID: 11929051]; PP1_strong:This variant shows co–segregation in the family [PMID: 28653968]	DM	0.83	–	0.0000070		
		c.439_440del	p.His147Glnfs*10	P	Frameshift deletion	PVS1, PM2_supporting, PS4_supporting:Two individuals with the mutation werw detected [PMID: 24997713]	DM		0.5 [spliceAI]	0.0000070		
		c.803G>A	p.Trp268*	P	Stopgain	PVS1, PM2_supporting, PS4_supporting:At least two individuals with the mutation werw detected [PMID: 10486317]	DM	0.69	–	0.0000070		
AR	ALAD (ADP)	c.823G>A	p.Val275Met	LP	Nonsynonymous SNV	PM2_supporting, PP3, PS3: This variant reduces enzyme activity [PMID: 11342419]; PM3: Conpound heterozygous variation with G133R was detected [PMID: 2063868]	DM	0.83	–	0.0000140	0.0003214(0.0002034–0.0004815) (1/3111)	0.00000002583092(0.00000001932658–0.00000003374837) (1/38713293)
		c.820G>A	p.Ala274Thr	LP	Nonsynonymous SNV	PM2_supporting, PP3, PS3: This variant reduces enzyme activity [PMID: 11342419]; PM3: Conpound heterozygous variation with R240W was detected [PMID: 11342419]	DM	0.81	–	0.0000070		
		c.718C>T	p.Arg240Trp	LP	Nonsynonymous SNV	PM2_supporting, PP3, PS3: This variant reduces enzyme activity [PMID: 11342419]; PM3: Conpound heterozygous variation with A274T was detected [PMID: 11342419]	DM	0.71	–	0.0000140		
		c.481+1G>T	–	LP	Splicing	PVS1, PM2_supporting			5 [ADA, RF, ssplice, maxent, spliceAI]	0.0000140		
		c.397G>A	p.Gly133Arg	LP	Nonsynonymous SNV	PM2_supporting, PP3, PS3: This variant reduces enzyme activity [PMID: 11342419]; PM3: Conpound heterozygous variation with V275M was detected [PMID: 2063868]	DM	0.84	3 [ADA, RF, maxent]	0.0000489		
		c.36C>G	p.Phe12Leu	LP	Nonsynonymous SNV	PM2_supporting, PS3: This variant reduces enzyme activity [PMID: 11342419]; PP1:This variant reduces enzyme activity [PMID: 10519994]	DM	0.66	–	0.0000419		
		c.691C>T	p.Arg231*	LP	Stopgain	PVS1, PM2_supporting			–	0.0000070		
		c.403delC	p.Leu135*	LP	Stopgain	PVS1, PM2_supporting			–	0.0000070		
		c.164+1G>T	–	LP	Splicing	PVS1, PM2_supporting			5 [ADA, RF, ssplice, maxent, spliceAI]	0.0000070		
	UROS (CEP)	c.244G>T	p.Val82Phe	LP	Nonsynonymous SNV	PM2_supporting, PP3, PS3: This variant affects enzyme activity [PMID: 7860775]; PM3: Conpound heterozygous with other variation was detected [PMID: 7860775]	DM	0.54	5 [ADA, RF, ssplice, maxent, spliceAI]	0.0000140	0.0009765(0.0007614–0.0012337) (1/1024)	0.00000023837830(0.00000017669647–0.00000031584471) (1/4195012)
		c.710T>C	p.Leu237Pro	LP	Nonsynonymous SNV	PM2_supporting, PP3, PS3: This variant affects enzyme activity [PMID: 19099412], PM3_supporting:Heterozygote individual of the mutation was detected [PMID: 17298225]	DM	0.94	–	0.0000070		
		c.607_608del	p.Tyr203Glnfs*11	LP	Frameshift deletion	PVS1, PM2_supporting			–	0.0000140		
		c.562–2A>G	–	LP	Splicing	PVS1, PM2_supporting			5 [ADA, RF, ssplice, maxent, spliceAI]	0.0000279		
		c.63+2T>C	–	LP	Splicing	PVS1, PM2_supporting	DM		4 [ADA, RF, maxent, spliceAI]	0.0000140		
		c.487G>T	p.Glu163*	LP	Stopgain	PVS1, PM2_supporting			1 [spliceAI]	0.0000070		
		c.42_43del	p.Cys14Trpfs*15	LP	Frameshift deletion	PVS1, PM2_supporting			–	0.0000070		
		c.673G>A	p.Gly225Ser	P	Nonsynonymous SNV	PM2_supporting, PP3, PS3: This variant affects enzyme activity [PMID: 7860775]; PM3_strong:Conpound heterozygous with other variation was detected [PMID: 7860775, 11254675, 19099412]	DM	0.95	–	0.0000628		
		c.63+1G>A	–	P	Splicing	PVS1, PM2_supporting, PM3: Conpound heterozygous with other variation was detected [PMID: 7860775]	DM		5 [ADA, RF, ssplice, maxent, spliceAI]	0.0000558		
		c.683C>T	p.Thr228Met	P	Nonsynonymous SNV	PM2_supporting, PP3, PS3: This variant affects enzyme activity [PMID: 1737856]; PM3_verystrong:Conpound heterozygous with other variation was detected, and detected homozygotes of this variation [PMID: 1737856, 19099412, 12060141, 22816431]	DM	0.95	–	0.0000349		
		c.217T>C	p.Cys73Arg	P	Nonsynonymous SNV	PM2_supporting, PP3, PS3: This variant affects enzyme activity [PMID: 1737856]; PM3_strong:Conpound heterozygous with other variation was detected, and detected homozygotes of this variation [PMID: 1737856, 19099412, 12060141]	DM	0.84	–	0.0002160		
		c.10C>T	p.Leu4Phe	P	Nonsynonymous SNV	PM2_supporting, PP3, PS3: This variant affects enzyme activity [PMID: 7860775]; PM3: Conpound heterozygous with other variation was detected [PMID: 7860775, 22816431]	DM	0.83	–	0.0000279		
	UROD (HEP)	c.346C>T	p.Gln116*	LP	Stopgain	PVS1, PM2_supporting	DM	0.02	–	0.0000070	0.0003239(0.0002039–0.0004827) (1/3087)	0.00000002623104(0.00000001942657–0.00000003388126) (1/38122773)
		c.842G>A	p.Gly281Glu	LP	Nonsynonymous SNV	PM2_supporting, PP3, PS3: The mutation reduced the enzyme activity, which was significantly lower in homozygote compared to the wild type [PMID: 8644733]; PM3: Heterozygote of the mutation was detected [PMID: 7706766]	DM	0.98	–	0.0000070		
		c.912C>A	p.Asn304Lys	LP	Nonsynonymous SNV	PM2_supporting, PP3, PS3: The mutation reduced the enzyme activity to 19.5% compared to the wild type [PMID: 9792863]	DM	0.79	–	0.0000070		
		c.397_398del	p.Tyr133Cysfs*34	LP	Frameshift deletion	PVS1, PM2_supporting	DM		–	0.0000070		
		c.424C>T	p.Arg142*	LP	Stopgain	PVS1, PM2_supporting	DM	0.11	–	0.0000070		
		c.616C>T	p.Gln206*	LP	Stopgain	PVS1, PM2_supporting	DM		1 [spliceAI]	0.0000070		
		c.649dupT	p.Glu218*	LP	Stopgain	PVS1, PM2_supporting	DM		2 [maxent, spliceAI]	0.0000070		
		c.1007A>G	p.Asn336Ser	LP	Nonsynonymous SNV	PM2_supporting, PP3, PS3: The mutation reduced the enzyme activity to 36.89% compared to the wild type [PMID: 17627795]	DM	0.93	–	0.0000279		
		c.1A>G	p.Met1?	LP	Startloss	PM2_supporting, PVS1_moderate, PP1_strong:This variant shows co–segregation in the family [PMID: 18462440]	DM	0.66	–	0.0000070		
		c.21–2A>C	–	LP	Canonical splicing	PVS1, PM2_supporting			3 [ADA, RF, spliceAI]	0.0000072		
		c.23_24insTCAG	p.Gly10Serfs*9	LP	Frameshift insertion	PVS1, PM2_supporting			–	0.0000070		
		c.27delG	p.Gly10Valfs*5	LP	Frameshift deletion	PVS1, PM2_supporting			–	0.0000071		
		c.30_31insCACCAGCTGCTTCGCATCCTCACTGATGCTCTGGTCCCATATCTGGTAGGACAAGTGGTGGC	p.Phe11Hisfs*25	LP	Frameshift insertion	PVS1, PM2_supporting			–	0.0000082		
		c.62delG	p.Arg21Glnfs*48	LP	Frameshift deletion	PVS1, PM2_supporting			1.5 [maxent, spliceAI]	0.0000279		
		c.820C>T	p.Gln274*	LP	Stopgain	PVS1, PM2_supporting		0.04	–	0.0000070		
		c.850T>C	p.Trp284Arg	LP	Nonsynonymous SNV	PM2_supporting, PP3, PS3: The mutation reduced the enzyme activity to 24% compared to the wild type [PMID: 23545314]; PS4_supporting:The variant was detected in 4 patients [PMID: 19233912, 23545314]	DM	0.45	–	0.0000070		
		c.494T>G	p.Met165Arg	P	Nonsynonymous SNV	PM2_supporting, PP3, PS3: The mutation reduced the enzyme activity to 1.36% compared to the wild type [PMID: 9792863]	DM	0.97	–	0.0000070		
	FECH (EPP)	c.820G>A	p.Asp274Asn	LP	Nonsynonymous SNV	PM2_supporting; PM3: Compound heterozygosity with multiple loci [PMID: 23364466, 16385445, 18787536]; PS3: The mutation reduced the enzyme activity to 0.8% compared to the wild type [PMID: 18787536]; PP3	DM	0.9	1 [maxent]	0.0000698	0.0007121(0.0005301–0.0009359) (1/1404)	0.00000012678144(0.00000008280194–0.00000018586113) (1/7887589)
		c.343C>T	p.Arg115*	LP	Stopgain	PVS1, PM2_supporting	DM		–	0.0000280		
		c.1001C>T	p.Pro334Leu	LP	Nonsynonymous SNV	PS3: The mutation reduced the enzyme activity to 19% compared to the wild type [PMID: 9585598]; PM2_supporting; PP3	DM	0.94	–	0.0000628		
		c.913G>T	p.Val305Phe	LP	Nonsynonymous SNV	PS3: The variant was shown to affect splicing by minigene experiments [PMID: 24586880]; PM2_supporting; PP3	DM	0.77	4 [ADA, RF, maxent, spliceAI]	0.0000209		
		c.901_902del	p.Trp301Alafs*23	LP	Frameshift deletion	PVS1, PM2_supporting	DM		1 [maxent]	0.0000070		
		c.580_584del	p.Tyr194Leufs*16	LP	Frameshift deletion	PVS1, PM2_supporting	DM		–	0.0000070		
		c.1078–1G>A	–	LP	Canonical splicing	PVS1, PM2_supporting	DM		4 [ADA, RF, maxent, spliceAI]	0.0000140		
		c.662G>A	p.Trp221*	LP	Stopgain	PVS1, PM2_supporting	DM		–	0.0000070		
		c.416A>T	p.Gln139Leu	LP	Nonsynonymous SNV	PM2_supporting, PP3; PM3_supporting:This variant was detected in a heterozygote individual [PMID: 15286165]; PS3: The mutation reduced the enzyme activity to 18% [PMID: 15286165]	DM	0.92	–	0.0000210		
		c.151C>T	p.Gln51*	LP	Stopgain	PVS1, PM2_supporting	DM		0.5 [spliceAI]	0.0000070		
		c.1077+1G>T	–	LP	Canonical splicing	PVS1, PM2_supporting			5 [ADA, RF, ssplice, maxent, spliceAI]	0.0000070		
		c.384delC	p.Gly129Aspfs*16	LP	Frameshift deletion	PVS1, PM2_supporting			–	0.0000070		
		c.378_382del	p.Ile126Metfs*22	LP	Frameshift deletion	PVS1, PM2_supporting			–	0.0000070		
		c.1217G>A	p.Cys406Tyr	LP	Nonsynonymous SNV	PM2_supporting, PP3; PS3: The mutation reduced the enzyme activity to 0.6%	DM	0.97	–	0.0000070		
		c.898G>T	p.Val300Leu	LP	Nonsynonymous SNV	PM2_supporting, PP3; PM3: Another suspected pathogenic variant was detected in another trans position [PMID: 18787536]; PS3: The mutation reduced the enzyme activity to 11% [PMID: 18787536]	DM	0.84	1 [maxent]	0.0000070		
		c.599–2A>G	–	LP	Canonical splicing	PVS1, PM2_supporting	DM		5 [ADA, RF, ssplice, maxent, spliceAI]	0.0000140		
		c.302T>C	p.Leu101Pro	LP	Nonsynonymous SNV	PM2_supporting, PP3; PM3: Another suspected pathogenic variant was detected in another trans position [PMID: 18787536]; PS3: The mutation reduced the enzyme activity to 5.3% [PMID: 18787536]	DM	0.95	–	0.0000070		
		c.757_761del	p.Ser254Glyfs*69	P	Frameshift deletion	PVS1, PM2_supporting, PP1:The variant was detected in two families [PMID: 24912412]	DM		–	0.0000210		
		c.314+2T>G	–	P	Canonical splicing	PVS1, PS3: RT–PCR results showed that this variant affects splicing [PMID: 8151124]	DM		4 [ADA, RF, maxent, spliceAI]	0.0000279		
		c.804+1G>A	–	P	Canonical splicing	PVS1, PM2_supporting, PS3: RT–PCR showed that this variant can lead to exon 7 jumps	DM		5 [ADA, RF, ssplice, maxent, spliceAI]	0.0000070		
		c.315–48T>C	–	VUS_P	Intronic	PS3: The mutation disrupts mRNA splicing [PMID: 11753383]; BA1	DFP		0.5 [spliceAI]	0.0628160	–	0.00002249555904 (0.0000005695–0.000125) (1/44453)

The asterisk in the table is for describing variation, means translation termination condon

Comparing the two databases showed that the distribution characteristics of AD porphyria in the Chinese population differed. Compared to the nine populations in gnomAD Genome V3.0, the predicted total carrier rates of all types of AD porphyria in the Chinese population were intermediate or low. The predicted carrier rate of pathogenic mutations in AIP patients followed the order of SAS > OTH > NFE > CHI > AFR > AMR > ASJ, with CHI ranking fourth. For PCT, the order was AMR > OTH > CHI > NFE > FIN > AFR, with CHI ranking third. For HCP, the order was AMI > OTH > SAS > NFE > AMR > AFR > CHI > FIN, with CHI ranking seventh. Last, for VP, the order was FIN > AFR > NFE > CHI, with CHI ranking last. The prevalence rates of AD hereditary porphyria among different ethnic populations in ChinaMAP and gnomAD Genome V3.0 are presented in Table 3.

**Table 3:** Predicted prevalence rates of porphyrias among different ethnicities in ChinaMAP and gnomAD

			CHI	EAS	ASJ	AMR	AFR	AMI
AD	HMBS (AIP)		0.0009445 (0.0004527–0.0017353) (1/1059)	0	0.0006017 (0.0000152–0.0033458) (1/1662)	0.0007324 (0.0002378–0017078) (1/1365)	0.0009040 (0.0005441–0.0014106) (1/1106)	0
	UROD (PCT)		0.0005664 (0.0002079–0.0012327) (1/1765)	0	0	0.0012148 (0.0005269–0.0024026) (1/825)	0.0001436 (0.0000296–0.0004196) (1/6963)	0
	CPOX (HCP)		0.0006611 (0.0002657–0.0013612) (1/1513)	0	0	0.0008796 (0.0003225–0.0019116) (1/1137)	0.0006662 (0.0003641–0.0011171) (1/1501)	0.00444 (0.0005375–0.0159267) (1/225)
	PPOX (VP)		0.0000944 (0.0000024–0.0005261) (1/10588)	0	0	0	0.000429 (0.0001961–0.0008139) (1/2331)	0
AR	ALAD (ADP)	Predict carrier rate	0.0001888 (0.0000229–0.0006821) (1/5294)	0	0	0.0001464 (0.0000037–0.0008154) (1/6830)	0.0007146 (0.0003993–0.0011762) (1/1400)	0
		Predict prevalence	0.00000000891136 (0.00000000247184–0.00000002276626) (1/112105744)	0	0	0.00000000535824 (0.00000000013570–0.00000002978302) (1/186595600)	0.00000012766329 (0.00000011127414–0.00000014515470) (1/7840000)	0
	UROS (CEP)	Predict carrier rate	0.0003776 (0.0001029–0.0009668) (1/2647)	0	0	0.0016086 (0.0008037–0.0028778) (1/621)	0.000808 (0.0004711–0.0012943) (1/1237)	0
		Predict prevalence	0.00000003564544 (0.00000002037835–0.00000005790729) (1/28026436)	0	0	0.00000064689849 (0.00000053914000–0.00000077642000) (1/1545837)	0.000000163216 (0.00000014539009–0.00000018373316) (1/6126850)	0
	UROD (HEP)	Predict carrier rate	0.0005664 (0.0002079–0.0012327) (1/1765)	0	0	0.0012148 (0.0005269–0.0024026) (1/825)	0.0001436 (0.0000296–0.0004196) (1/6963)	0
		Predict prevalence	0.00000008020224 (0.00000005624382–0.00000011122176) (1/12456194)	0	0	0.00000036893476 (0.00000028730806–0.00000047648296) (1/2722500)	0.00000000515524 (0.00000000240095–0.00000000976155) (1/193977389)	0
	FECH (EPP)	Predict carrier rate	0.0004722 (0.0001533–0.0011014) (1/2117)	0	0.0012037 (0.0001457–0.0043376) (1/830)	0	0.0005237 (0.0002615–0.0009369) (1/1909)	0
		Compound Heterozygous Prevalence of IVS-48-C-T	0.000075062929212 (0.00000190030653–0.00041812561438) (1/13322)	0	0.000038976175535 (0.00000098677926–0.00021713945629) (1/25656)	0	0.0000046561278168 (0.00000011788280–0.00002594192740) (1/214770)	0
		Predict prevalence	0.00007511868016 (0.00000190173394–0.00041843963362) (1/13312)	0	0.00003933841702 (0.00000099594018–0.00021915512459) (1/25420)	0	0.00000472477012 (0.00000011962054–0.00002632433967) (1/211650)	0

			FIN	NFE	SAS	OTH	gnomA D Genome V3.0
AD	HMBS (AIP)		0	0.0017347 (0.0013093–0.0022499) (1/577)	0.0032826 (0.0010654–0.0076346) (1/305)	0.0018579 (0.0002248–0.0066857) (1/538)	0.0012283 (0.0009846–0.0015121) (1/814)
	UROD (PCT)		0.000191 (0.0000048–0.0010638) (1/5234)	0.00031 (0.0001485–0.0005694) (1/3228)	0	0.000932 (0.0000236–0.0051767) (1/1073)	0.0003239 (0.0002039–0.0004827) (1/3087)
	CPOX (HCP)		0.0001914 (0.0000048–0.0010659) (1/5224)	0.001239 (0.0008848–0.0016858) (1/807)	0.00262 (0.0007137–0.0066889) (1/381)	0.00278 (0.0005737–0.0081037) (1/359)	0.0009769 (0.0007616–0.0012340) (1/1023)
	PPOX (VP)		0.001718 (0.0007851–0.0032555) (1/582)	0.000186 (0.0000682–0.0004044) (1/5381)	0	0	0.0003350 (0.0002147–0.0004986) (1/2985)
AR	ALAD (ADP)	Predict carrier rate	0	0.0002168 (0.0000872–0.0004466) (1/4612)	0	0	0.0003214 (0.0002034–0.0004815) (1/3111)
		Predict prevalence	0	0.00000001175056 (0.00000000392559–0.00000002798072) (1/85102327)	0	0	0.00000002583092 (0.00000001932658–0.00000003374837) (1/38713293)
	UROS (CEP)	Predict carrier rate	0	0.001177 (0.0008325–0.0016141) (1/849)	0.000656 (0.0000166–0.0036481 ) (1/1524)	0.002786 (0.0005743–0.0081112) (1/358)	0.0009765 (0.0007614–0.0012337) (1/1024)
		Predict prevalence	0	0.00000034633225 (0.00000029157762–0.00000040706601) (1/2887400)	0.000000107584 (0.00000000272697–0.00000060011909) (1/9290304)	0.000001940449 (0.00000089031019–0.00000369598866) (1/515344)	0.00000023837830 (0.00000017669647–0.00000031584471) (1/4195012)
	UROD (HEP)	Predict carrier rate	0.000191 (0.0000048–0.0010638) (1/5234)	0.00031 (0.0001485–0.0005694) (1/3228)	0	0.000932 (0.0000236–0.0051767) (1/1073)	0.0003239 (0.0002039–0.0004827) (1/3087)
		Predict prevalence	0.00000000912025 (0.00000000023109–0.00000005075580) (1/109579024)	0.000000024025 (0.00000001147198–0.00000004410644) (1/41679936)	0	0.000000217156 (0.00000000550264–0.00000121095387) (1/4605316)	0.00000002623104 (0.00000001942657–0.00000003388126) (1/38122773)
	FECH (EPP)	Predict carrier rate	0	0.0011769 (0.0008325–0.0016142) (1/849)	0.0006623 (0.0000168–0.0036818) (1/1510)	0	0.0007121 (0.0005301–0.0009359) (1/1404)
		Compound Heterozygous Prevalence of IVS-48-C-T	0	0.000027278698116 (0.00000069059216-.00015196792259) (1/36660)	0.000033308626592 (0.00000084328006–0.00018556504042) (1/30022)	0	0.0000224 (0.0000005671–0.000125) (1/44642)
		Predict prevalence	0	0.00002762498388 (0.00000069938672–0.00015390308250) (1/36199)	0.00003341827102 (0.00000084606995–0.00018617891342) (1/29923)	0	0.00002249555904 (0.0000005695–0.000125) (1/44453)

**Fig. 4:**
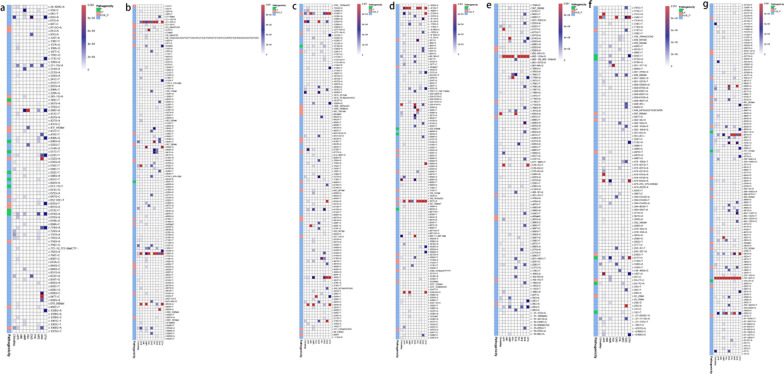
Distribution of P/LP and VUS-P mutations of porphyria-associated gene in different racial populations. (*Note* **a**–**g** P/LP and VUS-P mutations of *HMBS*, *UROD*, *CPOX*, *PPOX, ALAD*, *UROS*, and *FECH* in different ethnicities. The heatmap is arranged from top to bottom according to genomic location, with each column representing a population, drawing data from ChinaMAP and gnomAD Genome V3.0. Each cell in a row represents a locus, with a deeper color indicating a higher allele frequency in the population for that locus. The annotation panel on the far left indicates the rating of each locus: green for pathogenic, orange for likely pathogenic, and blue for uncertain significance.)

We compared all AD-inherited P/LP variant loci screened in the two databases and found that eight variants were specific to the Chinese population. These variants, namely, c.3G>A (p.Met1?), c.94C>T (p.Arg32Cys), c.422+1G>A, and c.499C>T (p.Arg167Trp) in *HMBS* and c.113dupA (p.Ala39Glyfs*9), c.213+1G>A, c.544dupT (p.Tyr182Leufs*7), and c.694delT (p.Phe232Leufs*13) in *UROD*, were included in ChinaMAP but not in gnomAD Genome V3.0. The c.1339C>T (p.Arg447Cys) variant of *CPOX* was the most widely distributed variant in both databases and was found in seven ethnic populations: CHI, AMR, AFR, AMI, NFE, SAS, and OTH. Additionally, ethnicity-specific AD-inherited P/LP variants were widely distributed in the two databases. The number and gene frequencies of AD hereditary P/LP gene variants in different ethnic populations in ChinaMAP and gnomAD Genome V3.0 are presented in Table 4. Furthermore, the distributions of P/LP and VUS-P mutations in different ethnic populations in gnomAD Genome V3.0 are shown in the graphs in Fig. 4a–d.

**Table 4:** Number and AF of porphyria-associated P/LP gene variants in different ethnicities in ChinaMAP and gnomAD

		DNA Change	AA Change	CHI		EAS		ASJ		AMR		AFR	
				Allele_Num	Allele_Frq	Allele_Num	Allele_Frq	Allele_Num	Allele_Frq	Allele_Num	Allele_Frq	Allele_Num	Allele_Frq
AD	HMBS (AIP)	c.3G>A	p.Met1?	1	0.0000472	–	–	–	–	–	–	–	–
		c.94C>T	p.Arg32Cys	1	0.0000472	–	–	–	–	–	–	–	–
		c.422+1G>A	–	2	0.0000944	–	–	–	–	–	–	–	–
		c.499C>T	p.Arg167Trp	1	0.0000472	–	–	–	–	–	–	–	–
		c.1064G>A	p.Arg355Gln	4	0.0001889	0	0	0	0	0	0	4	0.0000952
		c.91G>A	p.Ala31Thr	–	–	0	0	0	0	0	0	0	0
		c.345-1G>A	–	–	–	0	0	0	0	1	0.0000732	0	0
		c.347G>A	p.Arg116Gln	–	–	0	0	0	0	0	0	0	0
		c.457C>T	p.Gln153*	–	–	0	0	0	0	1	0.0000732	0	0
		c.532G>A	p.Asp178Asn	–	–	0	0	0	0	0	0	0	0
		c.655G>T	p.Asp178Asn	–	–	0	0	0	0	0	0	0	0
		c.754G>A	p.Ala252Thr	–	–	0	0	0	0	0	0	0	0
		c.992C>T	p.Ala331Val	–	–	0	0	0	0	0	0	2	0.0000476
		c.661G>A	p.Gly221Ser	–	–	0	0	0	0	0	0	1	0.0000238
		c.87+5G>A	–	–	–	0	0	0	0	0	0	0	0
		c.437_443del	p.Ser147Glufs*106	–	–	0	0	1	0.000300842	0	0	0	0
		c.647G>C	p.Gly216Ala	–	–	0	0	0	0	1	0.0000733	1	0.0000238
		c.674G>A	p.Arg225Gln	1	0.0000472	0	0	0	0	2	0.0001465	6	0.0001427
		c.346C>T	p.Arg116Trp	–	–	0	0	0	0	0	0	1	0.0000238
		c.500G>A	p.Arg167Gln	–	–	0	0	0	0	0	0	2	0.0000475
		c.517C>T	p.Arg173Trp	–	–	0	0	0	0	0	0	0	0
		c.583C>T	p.Arg195Cys	–	–	0	0	0	0	0	0	0	0
		c.601C>T	p.Arg201Trp	–	–	0	0	0	0	0	0	1	0.0000238
		c.613-1G>T	–	–	–	0	0	0	0	0	0	1	0.0000238
		c.673C>T	p.Arg225*	–	–	0	0	0	0	0	0	0	0
	UROD (PCT)	c.113dupA	p.Ala39Glyfs*9	2	0.0000944	–	–	–	–	–	–	–	–
		c.213+1G>A	–	1	0.0000472	–	–	–	–	–	–	–	–
		c.544dupT	p.Tyr182Leufs*7	1	0.0000472	–	–	–	–	–	–	–	–
		c.694delT	p.Phe232Leufs*13	2	0.0000944	–	–	–	–	–	–	–	–
		c.346C>T	p.Gln116*	–	–	0	0	0	0	0	0	1	0.0000238
		c.842G>A	p.Gly281Glu	–	–	0	0	0	0	1	0.0000732	0	0
		c.912C>A	p.Asn304Lys	–	–	0	0	0	0	1	0.0000732	0	0
		c.397_398del	p.Tyr133Cysfs*34	–	–	0	0	0	0	0	0	0	0
		c.424C>T	p.Arg142*	–	–	0	0	0	0	0	0	0	0
		c.616C>T	p.Gln206*	–	–	0	0	0	0	0	0	0	0
		c.649dupT	p.Glu218*	–	–	0	0	0	0	1	0.0000732	0	0
		c.1007A>G	p.Asn336Ser	–	–	0	0	0	0	0	0	1	0.0000238
		c.1A>G	p.Met1?	–	–	0	0	0	0	0	0	0	0
		c.21-2A>C	–	–	–	0	0	0	0	1	0.0000766	0	0
		c.23_24insTCAG	p.Gly10Serfs*9	–	–	0	0	0	0	0	0	0	0
		c.27delG	p.Gly10Valfs*5	–	–	0	0	0	0	0	0	1	0.0000242
		c.30_31insCACCAGCTGCTTCGCATCCTCACTGATGCTCTGGTCCCATATCTGGTAGGACAAGTGGTGGC	p.Phe11Hisfs*25	–	–	0	0	0	0	1	0.0000912	0	0
		c.62delG	p.Arg21Glnfs*48	–	–	0	0	0	0	3	0.0002200	0	0
		c.820C>T	p.Gln274*	–	–	0	0	0	0	0	0	0	0
		c.850T>C	p.Trp284Arg	–	–	0	0	0	0	0	0	0	0
		c.494T>G	p.Met165Arg	–	–	0	0	0	0	0	0	0	0
	CPOX (HCP)	c.1339C>T	p.Arg447Cys	6	0.0002833	0	0	0	0	3	0.00022	3	0.0000713
		c.1201C>T	p.Arg401Trp	1	0.0000472	0	0	0	0	0	0	0	0
		c.1277 + 3A>G	–	–	–	0	0	0	0	0	0	0	0
		c.877G>A	p.Ala293Thr	–	–	0	0	0	0	0	0	2	0.0000476
		c.601G>A	p.Glu201Lys	–	–	0	0	0	0	0	0	1	0.0000238
		c.131_132insGCAGC	p.Gly45Glnfs*93	–	–	0	0	0	0	1	0.0000734	1	0.0000238
		c.1006_1007insCC	p.Gly336Alafs*27	–	–	0	0	0	0	0	0	0	0
		c.1005_1006insC	p.Gly336Argfs*5	–	–	0	0	0	0	0	0	0	0
		c.997_1001del	p.Gly333Trpfs*6	–	–	0	0	0	0	0	0	0	0
		c.993delG	p.Arg332Glyfs*30	–	–	0	0	0	0	0	0	0	0
		c.953G>A	p.Trp318*	–	–	0	0	0	0	0	0	1	0.0000238
		c.916C>T	p.Gln306*	–	–	0	0	0	0	0	0	1	0.0000238
		c.688A>T	p.Lys230*	–	–	0	0	0	0	0	0	1	0.0000238
		c.663_667del	p.Met222Lysfs*9	–	–	0	0	0	0	1	0.0000732	0	0
		c.607G>A	p.Ala203Thr	–	–	0	0	0	0	0	0	0	0
		c.557-2A>G	–	–	–	0	0	0	0	0	0	1	0.0000238
		c.8delT	p.Leu3Cysfs*6	–	–	0	0	0	0	0	0	1	0.0000238
		c.1210A>G	p.Lys404Glu	–	–	0	0	0	0	1	0.0000732	2	0.0000476
	PPOX (VP)	c.1357G>A	p.Gly453Arg	1	0.0000472	0	0	0	0	0	0	1	0.0000238
		c.35T>C	p.Ile12Thr	–	–	0	0	0	0	0	0	0	0
		c.565C>T	p.Gln189*	–	–	0	0	0	0	0	0	0	0
		c.649C>T	p.Arg217Cys	–	–	0	0	0	0	0	0	0	0
		c.1072G>A	p.Gly358Arg	–	–	0	0	0	0	0	0	1	0.0000238
		c.532C>G	p.Leu178Val	–	–	0	0	0	0	0	0	0	0
		c.712C>T	p.Gln238*	–	–	0	0	0	0	0	0	1	0.0000238
		c.182dupT	p.Arg62*	–	–	0	0	0	0	0	0	1	0.0000238
		c.766_767insGG	p.Pro256Argfs*18	–	–	0	0	0	0	0	0	1	0.0000238
		c.767_768insT	p.Val257Glyfs*24	–	–	0	0	0	0	0	0	1	0.0000238
		c.1118G>A	p.Trp373*	–	–	0	0	0	0	0	0	1	0.0000238
		c.1123C>T	p.Gln375*	–	–	0	0	0	0	0	0	0	0
		c.1209_1210insATTTTTT	p.Lys404Ilefs*3	–	–	0	0	0	0	0	0	0	0
		c.1223delG	p.Ser408Thrfs*17	–	–	0	0	0	0	0	0	1	0.0000241
		c.503G>A	p.Arg168His	–	–	0	0	0	0	0	0	0	0
		c.439_440del	p.His147Glnfs*10	–	–	0	0	0	0	0	0	1	0.0000238
		c.803G>A	p.Trp268*	–	–	0	0	0	0	0	0	0	0
AR	ALAD (ADP)	c.458delT	p.Val153Glyfs*13	1	0.0000472	–	–	–	–	–	–	–	–
		c.823G>A	p.Val275Met	1	0.0000472	0	0	0	0	0	0	2	0.0000476
		c.820G>A	p.Ala274Thr	–	–	0	0	0	0	0	0	1	0.0000238
		c.718C>T	p.Arg240Trp	–	–	0	0	0	0	0	0	1	0.0000238
		c.481+1G>T	–	–	–	0	0	0	0	0	0	2	0.0000475
		c.397G>A	p.Gly133Arg	–	–	0	0	0	0	0	0	7	0.0001670
		c.36C>G	p.Phe12Leu	–	–	0	0	0	0	1	0.0000732	0	0
		c.691C>T	p.Arg231*	–	–	0	0	0	0	0	0	0	0
		c.403delC	p.Leu135*	–	–	0	0	0	0	0	0	1	0.0000238
		c.164+1G>T	–	–	–	0	0	0	0	0	0	1	0.0000238
	UROS (CEP)	c.588delT	p.Phe196Leufs*44	1	0.0000472	–	–	–	–	–	–	–	–
		c.320-2A>G	–	1	0.0000472	–	–	–	–	–	–	–	–
		c.244G>T	p.Val82Phe	–	–	0	0	0	0	0	0	0	0
		c.710T>C	p.Leu237Pro	–	–	0	0	0	0	0	0	0	0
		c.607_608del	p.Tyr203Glnfs*11	–	–	0	0	0	0	0	0	2	0.0000476
		c.562-2A>G	–	–	–	0	0	0	0	0	0	4	0.0000952
		c.63+2T>C	–	–	–	0	0	0	0	2	0.0001460	0	0
		c.487G>T	p.Glu163*	–	–	0	0	0	0	1	0.0000733	0	0
		c.42_43del	p.Cys14Trpfs*15	–	–	0	0	0	0	0	0	1	0.0000238
		c.673G>A	p.Gly225Ser	1	0.0000472	0	0	0	0	0	0	2	0.0000476
		c.63+1G>A	–	1	0.0000472	0	0	0	0	0	0	0	0
		c.683C>T	p.Thr228Met	–	–	0	0	0	0	2	0.0001460	1	0.0000238
		c.217T>C	p.Cys73Arg	–	–	0	0	0	0	6	0.0004390	7	0.0001660
		c.10C>T	p.Leu4Phe	–	–	0	0	0	0	0	0	0	0
	UROD (HEP)	c.113dupA	p.Ala39Glyfs*9	2	0.0000944	–	–	–	–	–	–	–	–
		c.213+1G>A	–	1	0.0000472	–	–	–	–	–	–	–	–
		c.544dupT	p.Tyr182Leufs*7	1	0.0000472	–	–	–	–	–	–	–	–
		c.694delT	p.Phe232Leufs*13	2	0.0000944	–	–	–	–	–	–	–	–
		c.346C>T	p.Gln116*	–	–	0	0	0	0	0	0	1	0.0000238
		c.842G>A	p.Gly281Glu	–	–	0	0	0	0	1	0.0000732	0	0
		c.912C>A	p.Asn304Lys	–	–	0	0	0	0	1	0.0000732	0	0
		c.397_398del	p.Tyr133Cysfs*34	–	–	0	0	0	0	0	0	0	0
		c.424C>T	p.Arg142*	–	–	0	0	0	0	0	0	0	0
		c.616C>T	p.Gln206*	–	–	0	0	0	0	0	0	0	0
		c.649dupT	p.Glu218*	–	–	0	0	0	0	1	0.0000732	0	0
		c.1007A>G	p.Asn336Ser	–	–	0	0	0	0	0	0	1	0.0000238
		c.1A>G	p.Met1?	–	–	0	0	0	0	0	0	0	0
		c.21-2A>C	–	–	–	0	0	0	0	1	0.0000766	0	0
		c.23_24insTCAG	p.Gly10Serfs*9	–	–	0	0	0	0	0	0	0	0
		c.27delG	p.Gly10Valfs*5	–	–	0	0	0	0	0	0	1	0.0000242
		c.30_31insCACCAGCTGCTTCGCATCCTCACTGATGCTCTGGTCCCATATCTGGTAGGACAAGTGGTGGC	p.Phe11Hisfs*25	–	–	0	0	0	0	1	0.0000912	0	0
		c.62delG	p.Arg21Glnfs*48	–	–	0	0	0	0	3	0.0002200	0	0
		c.820C>T	p.Gln274*	–	–	0	0	0	0	0	0	0	0
		c.850T>C	p.Trp284Arg	–	–	0	0	0	0	0	0	0	0
		c.494T>G	p.Met165Arg	–	–	0	0	0	0	0	0	0	0
	FECH (EPP)	c.924delG	p.Met308Ilefs*28	2	0.0000944	–	–	–	–	–	–	–	–
		c.820G>A	p.Asp274Asn	1	0.0000472	0	0	1	0.0003008	0	0	1	0.0000238
		c.343C>T	p.Arg115*	1	0.0000472	0	0	1	0.0003010	0	0	0	0
		c.1001C>T	p.Pro334Leu	–	–	0	0	0	0	0	0	1	0.0000238
		c.913G>T	p.Val305Phe	–	–	0	0	0	0	0	0	0	0
		c.901_902del	p.Trp301Alafs*23	–	–	0	0	0	0	0	0	0	0
		c.580_584del	p.Tyr194Leufs*16	–	–	0	0	0	0	0	0	0	0
		c.1078-1G>A	–	–	–	0	0	0	0	0	0	0	0
		c.662G>A	p.Trp221*	–	–	0	0	0	0	0	0	0	0
		c.416A>T	p.Gln139Leu	–	–	0	0	0	0	0	0	1	0.0000238
		c.151C>T	p.Gln51*	–	–	0	0	0	0	0	0	1	0.0000238
		c.1077+1G>T	–	–	–	0	0	0	0	0	0	0	0
		c.384delC	p.Gly129Aspfs*16	–	–	0	0	0	0	0	0	1	0.0000238
		c.378_382del	p.Ile126Metfs*22	–	–	0	0	0	0	0	0	1	0.0000238
		c.1217G>A	p.Cys406Tyr	–	–	0	0	0	0	0	0	1	0.0000238
		c.898G>T	p.Val300Leu	–	–	0	0	0	0	0	0	0	0
		c.599-2A>G	–	–	–	0	0	0	0	0	0	0	0
		c.302T>C	p.Leu101Pro	–	–	0	0	0	0	0	0	0	0
		c.757_761del	p.Ser254Glyfs*69	1	0.0000472	0	0	0	0	0	0	3	0.0000715
		c.314+2T>G	–	–	–	0	0	0	0	0	0	0	0
		c.804+1G>A	–	–	–	0	0	0	0	0	0	0	0
		c.315-48T>C	–	6732	0.317907	1000	0.319898	215	0.064759	2453	0.179654	822	0.0195528

		DNA Change	AA Change	AMI		FIN		NFE		SAS		OTH	
				Allele_Num	Allele_Frq	Allele_Num	Allele_Frq	Allele_Num	Allele_Frq	Allele_Num	Allele_Frq	Allele_Num	Allele_Frq
AD	HMBS (AIP)	c.3G>A	p.Met1?	–	–	–	–	–	–	–	–	–	–
		c.94C>T	p.Arg32Cys	–	–	–	–	–	–	–	–	–	–
		c.422+1G>A	–	–	–	–	–	–	–	–	–	–	–
		c.499C>T	p.Arg167Trp	–	–	–	–	–	–	–	–	–	–
		c.1064G>A	p.Arg355Gln	0	0	0	0	4	0.0000620	1	0.000328947	0	0
		c.91G>A	p.Ala31Thr	0	0	0	0	1	0.0000155	0	0	0	0
		c.345-1G>A	–	0	0	0	0	0	0	0	0	0	0
		c.347G>A	p.Arg116Gln	0	0	0	0	2	0.0000310	0	0	0	0
		c.457C>T	p.Gln153*	0	0	0	0	0	0	0	0	0	0
		c.532G>A	p.Asp178Asn	0	0	0	0	0	0	4	0.00131234	0	0
		c.655G>T	p.Asp178Asn	0	0	0	0	5	0.0000774	0	0	1	0.000464684
		c.754G>A	p.Ala252Thr	0	0	0	0	2	0.0000310	0	0	0	0
		c.992C>T	p.Ala331Val	0	0	0	0	4	0.0000620	0	0	0	0
		c.661G>A	p.Gly221Ser	0	0	0	0	0	0	0	0	0	0
		c.87+5G>A	–	0	0	0	0	1	0.0000155	0	0	0	0
		c.437_443del	p.Ser147Glufs*106	0	0	0	0	0	0	0	0	0	0
		c.647G>C	p.Gly216Ala	0	0	0	0	0	0	0	0	0	0
		c.674G>A	p.Arg225Gln	0	0	0	0	23	0.0003563	0	0	1	0.000464253
		c.346C>T	p.Arg116Trp	0	0	0	0	0	0	0	0	0	0
		c.500G>A	p.Arg167Gln	0	0	0	0	7	0.0001084	0	0	0	0
		c.517C>T	p.Arg173Trp	0	0	0	0	1	0.0000155	0	0	0	0
		c.583C>T	p.Arg195Cys	0	0	0	0	1	0.0000155	0	0	0	0
		c.601C>T	p.Arg201Trp	0	0	0	0	4	0.0000619	0	0	0	0
		c.613-1G>T	–	0	0	0	0	0	0	0	0	0	0
		c.673C>T	p.Arg225*	0	0	0	0	1	0.0000155	0	0	0	0
	UROD (PCT)	c.113dupA	p.Ala39Glyfs*9	–	–	–	–	–	–	–	–	–	–
		c.213+1G>A	–	–	–	–	–	–	–	–	–	–	–
		c.544dupT	p.Tyr182Leufs*7	–	–	–	–	–	–	–	–	–	–
		c.694delT	p.Phe232Leufs*13	–	–	–	–	–	–	–	–	–	–
		c.346C>T	p.Gln116*	0	0	0	0	0	0	0	0	0	0
		c.842G>A	p.Gly281Glu	0	0	0	0	0	0	0	0	0	0
		c.912C>A	p.Asn304Lys	0	0	0	0	0	0	0	0	0	0
		c.397_398del	p.Tyr133Cysfs*34	0	0	0	0	1	0.0000155	0	0	0	0
		c.424C>T	p.Arg142*	0	0	0	0	1	0.0000155	0	0	0	0
		c.616C>T	p.Gln206*	0	0	0	0	1	0.0000155	0	0	0	0
		c.649dupT	p.Glu218*	0	0	0	0	0	0	0	0	0	0
		c.1007A>G	p.Asn336Ser	0	0	0	0	3	0.0000465	0	0	0	0
		c.1A>G	p.Met1?	0	0	0	0	1	0.0000155	0	0	0	0
		c.21-2A>C	–	0	0	0	0	0	0	0	0	0	0
		c.23_24insTCAG	p.Gly10Serfs*9	0	0	1	0.0000955	0	0	0	0	0	0
		c.27delG	p.Gly10Valfs*5	0	0	0	0	0	0	0	0	0	0
		c.30_31insCACCAGCTGCTTCGCATCCTCACTGATGCTCTGGTCCCATATCTGGTAGGACAAGTGGTGGC	p.Phe11Hisfs*25	0	0	0	0	0	0	0	0	0	0
		c.62delG	p.Arg21Glnfs*48	0	0	0	0	0	0	0	0	1	0.000466
		c.820C>T	p.Gln274*	0	0	0	0	1	0.0000155	0	0	0	0
		c.850T>C	p.Trp284Arg	0	0	0	0	1	0.0000155	0	0	0	0
		c.494T>G	p.Met165Arg	0	0	0	0	1	0.0000155	0	0	0	0
	CPOX (HCP)	c.1339C>T	p.Arg447Cys	2	0.00222	0	0	24	0.000372	4	0.00131	3	0.00139
		c.1201C>T	p.Arg401Trp	0	0	1	0.0000957	0	0	0	0	0	0
		c.1277 + 3A>G	–	0	0	0	0	3	0.0000465	0	0	0	0
		c.877G>A	p.Ala293Thr	0	0	0	0	0	0	0	0	0	0
		c.601G>A	p.Glu201Lys	0	0	0	0	3	0.0000465	0	0	0	0
		c.131_132insGCAGC	p.Gly45Glnfs*93	0	0	0	0	0	0	0	0	0	0
		c.1006_1007insCC	p.Gly336Alafs*27	0	0	0	0	0	0	0	0	0	0
		c.1005_1006insC	p.Gly336Argfs*5	0	0	0	0	0	0	0	0	0	0
		c.997_1001del	p.Gly333Trpfs*6	0	0	0	0	0	0	0	0	0	0
		c.993delG	p.Arg332Glyfs*30	0	0	0	0	0	0	0	0	0	0
		c.953G>A	p.Trp318*	0	0	0	0	0	0	0	0	0	0
		c.916C>T	p.Gln306*	0	0	0	0	0	0	0	0	0	0
		c.688A>T	p.Lys230*	0	0	0	0	0	0	0	0	0	0
		c.663_667del	p.Met222Lysfs*9	0	0	0	0	0	0	0	0	0	0
		c.607G>A	p.Ala203Thr	0	0	0	0	1	0.0000155	0	0	0	0
		c.557-2A>G	–	0	0	0	0	0	0	0	0	0	0
		c.8delT	p.Leu3Cysfs*6	0	0	0	0	0	0	0	0	0	0
		c.1210A>G	p.Lys404Glu	0	0	0	0	9	0.000139	0	0	0	0
	PPOX (VP)	c.1357G>A	p.Gly453Arg	0	0	0	0	0	0	0	0	0	0
		c.35T>C	p.Ile12Thr	0	0	7	0.0006680	0	0	0	0	0	0
		c.565C>T	p.Gln189*	0	0	0	0	1	0.0000155	0	0	0	0
		c.649C>T	p.Arg217Cys	0	0	2	0.0001910	1	0.0000155	0	0	0	0
		c.1072G>A	p.Gly358Arg	0	0	0	0	0	0	0	0	0	0
		c.532C>G	p.Leu178Val	0	0	0	0	1	0.0000155	0	0	0	0
		c.712C>T	p.Gln238*	0	0	0	0	0	0	0	0	0	0
		c.182dupT	p.Arg62*	0	0	0	0	0	0	0	0	0	0
		c.766_767insGG	p.Pro256Argfs*18	0	0	0	0	0	0	0	0	0	0
		c.767_768insT	p.Val257Glyfs*24	0	0	0	0	0	0	0	0	0	0
		c.1118G>A	p.Trp373*	0	0	0	0	0	0	0	0	0	0
		c.1123C>T	p.Gln375*	0	0	0	0	1	0.0000155	0	0	0	0
		c.1209_1210insATTTTTT	p.Lys404Ilefs*3	0	0	0	0	0	0	0	0	0	0
		c.1223delG	p.Ser408Thrfs*17	0	0	0	0	0	0	0	0	0	0
		c.503G>A	p.Arg168His	0	0	0	0	1	0.0000155	0	0	0	0
		c.439_440del	p.His147Glnfs*10	0	0	0	0	0	0	0	0	0	0
		c.803G>A	p.Trp268*	0	0	0	0	1	0.0000155	0	0	0	0
AR	ALAD (ADP)	c.458delT	p.Val153Glyfs*13	–	–	–	–	–	–	–	–	–	–
		c.823G>A	p.Val275Met	0	0	0	0	0	0	0	0	0	0
		c.820G>A	p.Ala274Thr	0	0	0	0	0	0	0	0	0	0
		c.718C>T	p.Arg240Trp	0	0	0	0	1	0.0000155	0	0	0	0
		c.481+1G>T	–	0	0	0	0	0	0	0	0	0	0
		c.397G>A	p.Gly133Arg	0	0	0	0	0	0	0	0	0	0
		c.36C>G	p.Phe12Leu	0	0	0	0	5	0.0000774	0	0	0	0
		c.691C>T	p.Arg231*	0	0	0	0	1	0.0000155	0	0	0	0
		c.403delC	p.Leu135*	0	0	0	0	0	0	0	0	0	0
		c.164+1G>T	–	0	0	0	0	0	0	0	0	0	0
	UROS (CEP)	c.588delT	p.Phe196Leufs*44	–	–	–	–	–	–	–	–	–	–
		c.320-2A>G	–	–	–	–	–	–	–	–	–	–	–
		c.244G>T	p.Val82Phe	0	0	0	0	2	0.0000310	0	0	0	0
		c.710T>C	p.Leu237Pro	0	0	0	0	0	0	1	0.0003280	0	0
		c.607_608del	p.Tyr203Glnfs*11	0	0	0	0	0	0	0	0	0	0
		c.562-2A>G	–	0	0	0	0	0	0	0	0	0	0
		c.63+2T>C	–	0	0	0	0	0	0	0	0	0	0
		c.487G>T	p.Glu163*	0	0	0	0	0	0	0	0	0	0
		c.42_43del	p.Cys14Trpfs*15	0	0	0	0	0	0	0	0	0	0
		c.673G>A	p.Gly225Ser	0	0	0	0	7	0.0001080	0	0	0	0
		c.63+1G>A	–	0	0	0	0	8	0.0001240	0	0	0	0
		c.683C>T	p.Thr228Met	0	0	0	0	1	0.0000155	0	0	1	0.0004640
		c.217T>C	p.Cys73Arg	0	0	0	0	16	0.0002480	0	0	2	0.0009290
		c.10C>T	p.Leu4Phe	0	0	0	0	4	0.0000620	0	0	0	0
	UROD (HEP)	c.113dupA	p.Ala39Glyfs*9	–	–	–	–	–	–	–	–	–	–
		c.213+1G>A	–	–	–	–	–	–	–	–	–	–	–
		c.544dupT	p.Tyr182Leufs*7	–	–	–	–	–	–	–	–	–	–
		c.694delT	p.Phe232Leufs*13	–	–	–	–	–	–	–	–	–	–
		c.346C>T	p.Gln116*	0	0	0	0	0	0	0	0	0	0
		c.842G>A	p.Gly281Glu	0	0	0	0	0	0	0	0	0	0
		c.912C>A	p.Asn304Lys	0	0	0	0	0	0	0	0	0	0
		c.397_398del	p.Tyr133Cysfs*34	0	0	0	0	1	0.0000155	0	0	0	0
		c.424C>T	p.Arg142*	0	0	0	0	1	0.0000155	0	0	0	0
		c.616C>T	p.Gln206*	0	0	0	0	1	0.0000155	0	0	0	0
		c.649dupT	p.Glu218*	0	0	0	0	0	0	0	0	0	0
		c.1007A>G	p.Asn336Ser	0	0	0	0	3	0.0000465	0	0	0	0
		c.1A>G	p.Met1?	0	0	0	0	1	0.0000155	0	0	0	0
		c.21-2A>C	–	0	0	0	0	0	0	0	0	0	0
		c.23_24insTCAG	p.Gly10Serfs*9	0	0	1	0.0000955	0	0	0	0	0	0
		c.27delG	p.Gly10Valfs*5	0	0	0	0	0	0	0	0	0	0
		c.30_31insCACCAGCTGCTTCGCATCCTCACTGATGCTCTGGTCCCATATCTGGTAGGACAAGTGGTGGC	p.Phe11Hisfs*25	0	0	0	0	0	0	0	0	0	0
		c.62delG	p.Arg21Glnfs*48	0	0	0	0	0	0	0	0	1	0.0004660
		c.820C>T	p.Gln274*	0	0	0	0	1	0.0000155	0	0	0	0
		c.850T>C	p.Trp284Arg	0	0	0	0	1	0.0000155	0	0	0	0
		c.494T>G	p.Met165Arg	0	0	0	0	1	0.0000155	0	0	0	0
	FECH (EPP)	c.924delG	p.Met308Ilefs*28	–	–	–	–	–	–	–	–	–	–
		c.820G>A	p.Asp274Asn	0	0	0	0	8	0.0001239	0	0	0	0
		c.343C>T	p.Arg115*	0	0	0	0	2	0.0000310	1	0.0003311	0	0
		c.1001C>T	p.Pro334Leu	0	0	0	0	8	0.0001239	0	0	0	0
		c.913G>T	p.Val305Phe	0	0	0	0	3	0.0000465	0	0	0	0
		c.901_902del	p.Trp301Alafs*23	0	0	0	0	1	0.0000155	0	0	0	0
		c.580_584del	p.Tyr194Leufs*16	0	0	0	0	1	0.0000155	0	0	0	0
		c.1078-1G>A	–	0	0	0	0	2	0.0000310	0	0	0	0
		c.662G>A	p.Trp221*	0	0	0	0	1	0.0000155	0	0	0	0
		c.416A>T	p.Gln139Leu	0	0	0	0	2	0.0000310	0	0	0	0
		c.151C>T	p.Gln51*	0	0	0	0	0	0	0	0	0	0
		c.1077+1G>T	–	0	0	0	0	1	0.0000155	0	0	0	0
		c.384delC	p.Gly129Aspfs*16	0	0	0	0	0	0	0	0	0	0
		c.378_382del	p.Ile126Metfs*22	0	0	0	0	0	0	0	0	0	0
		c.1217G>A	p.Cys406Tyr	0	0	0	0	0	0	0	0	0	0
		c.898G>T	p.Val300Leu	0	0	0	0	1	0.0000155	0	0	0	0
		c.599-2A>G	–	0	0	0	0	2	0.0000310	0	0	0	0
		c.302T>C	p.Leu101Pro	0	0	0	0	1	0.0000155	0	0	0	0
		c.757_761del	p.Ser254Glyfs*69	0	0	0	0	0	0	0	0	0	0
		c.314+2T>G	–	0	0	0	0	4	0.0000619	0	0	0	0
		c.804+1G>A	–	0	0	0	0	1	0.0000155	0	0	0	0
		c.315-48T>C	–	1	0.00111111	1017	0.0974325	2992	0.0463560	306	0.100592	190	0.0884544

The asterisk in the table is for describing variation, means translation termination condon

### AR porphyria-related gene variants

#### Characteristics of P/LP variants in AR-inherited genes in ChinaMAP

In the ChinaMAP study, a total of 14 AR-inherited P/LP variants were screened, including 2 variants in *ALAD*, 4 in *UROS*, 4 in *UROD*, and 4 in *FECH*. *ALAD* exhibited the least variety among variant loci. The predicted carrier rates of pathogenic variants for each type of AR porphyria in the Chinese population were as follows: ADP, 1/5294 (1.888 × 10^−4^, 2.29 × 10^−5^–6.821 × 10^−4^); CEP, 1/2647 (3.776 × 10^−4^, 1.029 × 10^−4^–9.668 × 10^−4^); HEP, 1/1765 (5.664 × 10^−4^, 2.079 × 10^−4^–1.2327 × 10^−3^); and EPP, 1/2117 (4.722 × 10^−4^, 1.533 × 10^−4^–1.101 × 10^−3^). The predicted prevalence of each type of AR porphyria in the Chinese population was as follows: ADP, 8.91 × 10^−9^ (8.91 × 10^−9^, 2.472 × 10^−9^–2.277 × 10^−8^); CEP, 3.565 × 10^−8^ (3.565 × 10^−8^, 2.038 × 10^−8^–5.791 × 10^−8^); and HEP, 8.02 × 10^−8^ (8.02 × 10^−8^, 5.624 × 10^−8^–1.112 × 10^−7^). The AF of the *FECH* low-expression SNP locus c.315-48T>C in the Chinese population was 0.317907, and the predicted prevalence of EPP was 7.51 × 10^−5^ (7.51 × 10^−5^, 1.902 × 10^−6^–4.184 × 10^−4^). The predicted carrier rate for HEP was the greatest among the AR porphyrias, while ADP had the lowest carrier rate. EPP was predicted to have the highest prevalence, while ADP had the lowest prevalence. Missense mutations were found to be the most common type of variant among the AR porphyrias. Table 1 includes information for all AR-inherited porphyria-associated P/LP variant loci and the *FECH* low-expression SNP locus c.315-48T>C in ChinaMAP. Additionally, the charts in Fig. 2d and g–i contain information about P/LP and VUS-P variations in various genes.

### Distribution and characteristics of AR-inherited P/LP variants in different ethnic populations

In accordance with the ACMG guidelines, a total of 58 AR-inherited P/LP variants were examined in gnomAD Genome V3.0. Of these, 9 were identified in *ALAD*, 12 in *UROS*, 17 in *UROD*, and 20 in *FECH*. Notably, *FECH* had the greatest number of loci with variants, whereas *ALAD* had the lowest number. The carrier rates of predicted pathogenic mutations for ADP in ChinaMAP and gnomAD were 1/5294 and 1/3111, respectively. For CEP, the rates in ChinaMAP and gnomAD were 1/2647 and 1/1024, respectively. For HEP, the rates in ChinaMAP and gnomAD were 1/1765 and 1/3087, respectively. For EPP, the rates in ChinaMAP and gnomAD were 1/2117 and 1/1404, respectively. The predicted prevalence of ADP was 8.91 × 10^−9^ in ChinaMAP and 2.58 × 10^−8^ in gnomAD. The predicted prevalence of CEP was 3.565 × 10^−8^ in ChinaMAP and 2.38 × 10^−7^ in gnomAD. Similarly, the predicted prevalence of HEP was 8.02 × 10^−8^ in ChinaMAP and 2.623 × 10^−8^ in gnomAD. Finally, the predicted prevalence of EPP was 7.51 × 10^−5^ in ChinaMAP and 2.25 × 10^−5^ in gnomAD. Missense mutations were the most frequent type of variant in these loci. Table 2 displays the gnomAD Genome V3.0 information for all AR-inherited porphyria-associated P/LP variant loci and the *FECH* low-expression SNP locus c.315-48T>C. The graphs in Fig. 3b and e–g display information for the P/LP and VUS-P variants in each gene.

The distribution characteristics of AR porphyria in the Chinese population were compared with those of the 9 populations in gnomAD Genome V3.0. The predicted carrier rate and prevalence for ADP were ranked as AFR > NFE > CHI > AMR, with CHI ranking third. For CEP, the predicted carrier rate and prevalence rate were ranked as OTH > AMR > NFE > AFR > SAS > CHI, with CHI ranking last. For HEP, the predicted carrier rates were ranked as AMR > OTH > CHI > NFE > FIN > AFR, with the Chinese population ranking third. For EPP, the predicted carrier rates were ranked as ASJ > NFE > SAS > AFR > CHI, with CHI ranking last, while the prevalence rates were ranked as CHI > ASJ > SAS > NFE > AFR, with CHI ranking first. Table 3 displays the anticipated carrier rates and prevalence rates of AR porphyria among various ethnic populations in ChinaMAP and gnomAD Genome V3.0.

Comparing the distribution of all AR P/LP variant loci screened in the two databases across different populations showed that eight variants were unique to the Chinese population. These variants, including c.458delT (p.Val153Glyfs*13) in *ALAD*; c.924delG (p.Met308Ilefs*28) in *FECH*; c.113dupA (p.Ala39Glyfs*9), c.213+1G>A, c.544dupT (p.Tyr182Leufs*7), and c.694delT (p.Phe232Leufs*13) in *UROD*; and c.588delT (p.Phe196Leufs*44) and c.320-2A>G in *UROS*, were included in ChinaMAP but not in gnomAD Genome V3.0. The *FECH* low-expression SNP locus c.315-48T>C was found in all 10 populations in the two databases, ranked in the order of EAS > CHI > AMR > SAS > FIN > OTH > ASJ > NFE > AFR > AMI. Additionally, ethnicity-specific AR genetic P/LP variants were widely distributed in both databases. Table 4 presents the number and AFs of AR P/LP variants and the *FECH* low-expression SNP locus c.315-48T>C in different ethnic populations in ChinaMAP and gnomAD Genome V3.0. The graphs in Fig. 4b and e–g depict the distribution of P/LP and VUS-P variants of each gene in various ethnic populations in the gnomAD Genome V3.0 database.

### X-linked inherited porphyria-related genetic variants

The X-linked inherited P/LP variant of *ALAS2* was not found in ChinaMAP; therefore, no ACMG ratings were obtained for this gene in this study, and no XLP prevalence prediction was performed.

## Discussion

The distribution of P/LP variants and the carrier and prevalence rates of each type of porphyria vary by ethnicity due to genetic heterogeneity, making its assessment complex. Current data on the genetics of porphyria come mainly from individual countries in Europe and the Japanese population in Asia. Data from large-scale population-based genetic studies in these regions are lacking, with reports limited to case reports, small group studies, and family studies. The limited diagnosis, treatment, and genetic research on porphyria within the Chinese medical system have resulted in a high rate of clinical misdiagnosis and posed challenges in treatment, sometimes endangering the patient's life.

Studies on AD porphyria have produced various findings. Grandchamp B's review on AIP suggests that asymptomatic heterozygotes for the AIP gene variants may have a prevalence of approximately 1/2000 [7], while Hugo Lenglet states that the lowest estimate of the prevalence of AIP in the general population is 1/1299 [8]. The prevalence of AIP is extremely low, with a prevalence of approximately 0.5–1% in the general population [8]. The predicted AIP gene mutations prevalence in France is 1/1675 [9], 5.9/1,000,000 in Europe [10], and 1.5/100,000 in Japan [11]. It has also been reported that the prevalence of symptomatic European AIP heterozygotes is approximately 0.000005, and the penetrance of acute attacks is about 1% [12]. Our team’s previous findings also predicted that the prevalence of the pathogenic *HMBS* variant in the Chinese population was 1/1765 [13]. PCT is the most prevalent type of porphyria in Europe, with a prevalence of 1/10,000 [14]. The estimated prevalence of HCP in Europe is 0.2/10,000,000 [10]. HCP is more prevalent in the South African population, with a prevalence of approximately 1/100000 [15], while VP is rarer in Europe, with a prevalence of 3.2/1,000,000 [10]. The prevalence of VP in Finland is 2.4/1,000,000 [10].

Regarding AR porphyria, the overall prevalence of ADP, CEP, and HEP is 0.13/10000000, with CEP accounting for more than half [10]. The prevalence of EPP varies significantly among different populations, largely due to the influence of the low-expression allele c.315-48T>C. EPP has a worldwide prevalence ranging from 1/75,000 to 1/200,000 [16], with a prevalence of 9.2/1,000,000 in Europe [10].

In this study, we utilized the ChinaMAP genetic database, a reliable and scientific database for the Chinese population. This is the first extensive genetic study of porphyria in the Chinese population, offering reliable reference data for genetic screening, preventive interventions, early diagnosis, and the management of patients with latent porphyria in China. Simultaneously, an analysis of genetic data on porphyria in the Chinese population was conducted, and the results were compared with those of other ethnic groups to gain a better understanding of its distinct characteristics. This study can serve as a valuable reference for porphyria-related research in the Chinese population.

**Fig. 5:**
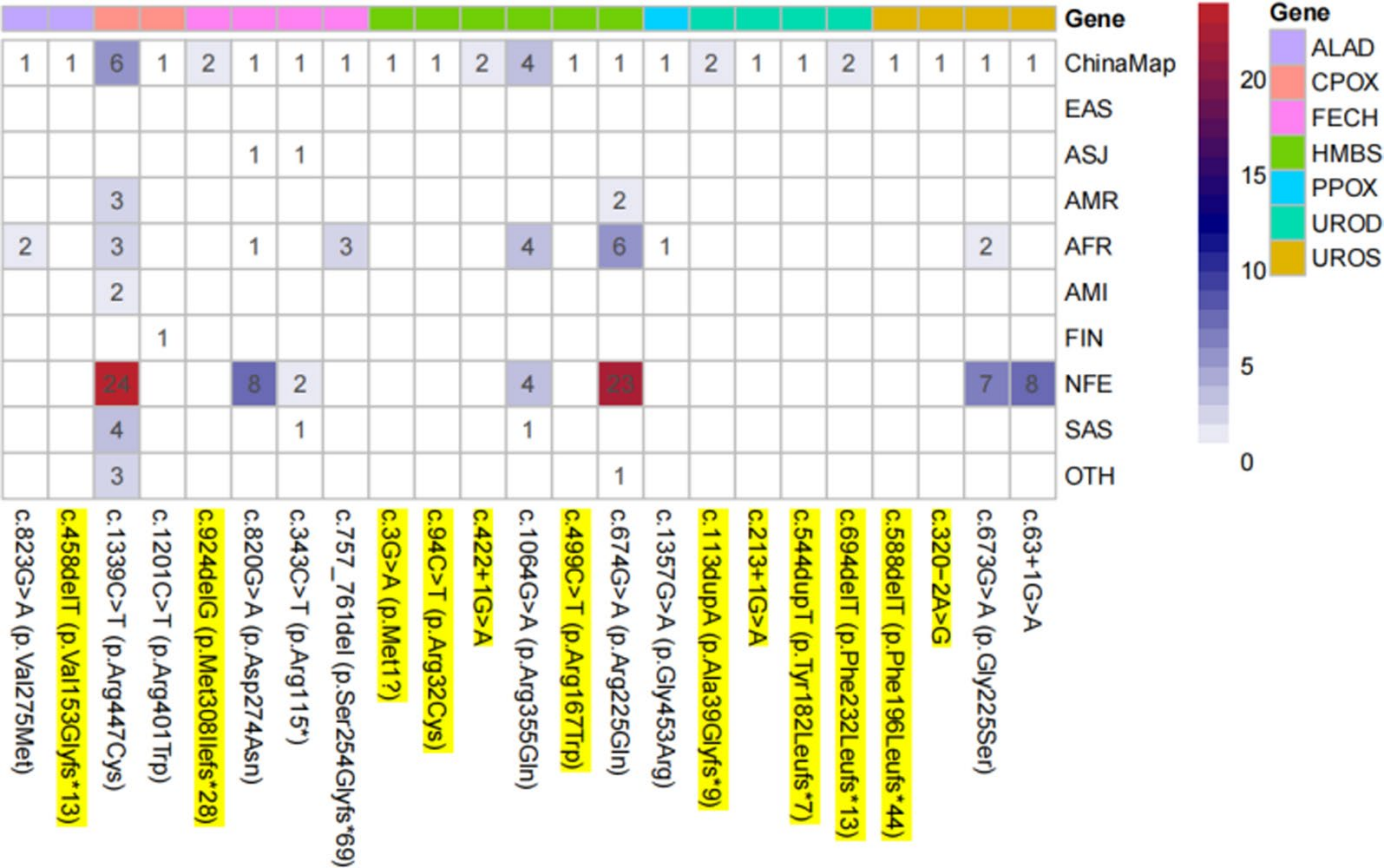
Distribution of porphyria-associated gene P/LP variant loci in ChinaMAP in different ethnic populations. (*Note* The reference sequences for *ALAD* DNA and protein are RefSeq NM_000031.6 and NP_000022.3, respectively; for *CPOX* DNA and protein are RefSeq NM_000097.7 and NP_000088.3, respectively; for *FECH* DNA and protein are RefSeq NM_ 000031.6 and NP_000022.3; the reference sequences of *HMBS* DNA and protein are RefSeq NM_000190.4 and NP_000181.2; the reference sequences of *PPOX* DNA and protein are RefSeq NM_000309.5 and NP_000300.1; the reference sequences of *UROD* DNA and protein are RefSeq NM_000309.5 and NP_000300.1; The reference sequences of UROD DNA and protein are RefSeq NM_000374.5 and NP_000365.3, respectively; the reference sequences of *UROS* DNA and protein are RefSeq NM_000375.3 and NP_000366.1, respectively; P/LP variants specific to the Chinese population are highlighted in yellow. The blue-red color code indicates the number of each porphyria-related gene P/LP variation loci, the greater the redder.)

In ChinaMAP, a total of 23 P/LP porphyria-associated genetic variants were identified in seven genes. The predicted carrier and prevalence rates for each porphyria type in the Chinese population were then calculated based on HWE. The predicted prevalence of EPP in the Chinese population was the highest among the 10 ethnic groups, whereas the predicted carrier and prevalence rates of the other porphyrias were moderate or low. We found 12 P/LP variants in porphyria-associated genes that are specific to the Chinese population in comparison to gnomAD Genome V3.0. In our previous study, we classified the *HMBS* c.1064G>A (p.Arg355Gln) locus as a VUS-P. However, in our current study, after reviewing recent literature, we found that Hugo Lenglet confirmed that the presence of this locus resulted in almost no HMBS activity. As a result, we added PS3 evidence for this locus according to the ACMG guidelines and upgraded its classification to LP in this study. Figure 5 illustrates the distribution of P/LP variant sites of porphyria-related genes in ChinaMAP across the 10 populations studied. These results showed that the variant profiles of porphyria-associated genes differ between the Chinese population and other ethnic groups.

**Fig. 6:**
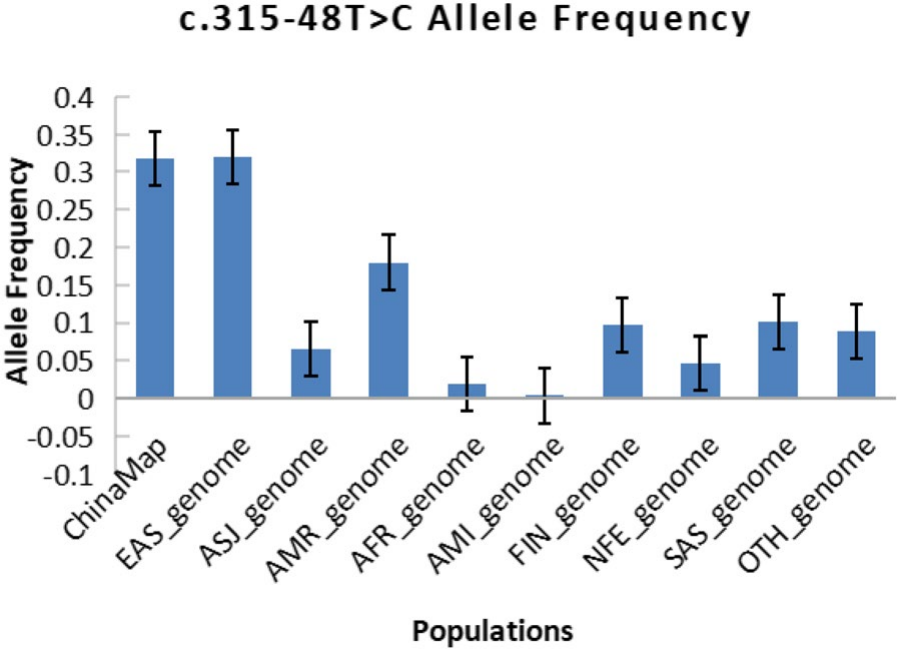
Population frequency of the FECH low-expression SNP locus c.315-48T>C. (*Note* The error bars represent the mean plus or minus the standard error.)

When comparing the AF of the *FECH* low-expression SNP locus c.315-48T>C in different ethnic populations, the Chinese population had the second highest frequency. Figure 6 displays the distribution of this locus among the various ethnic groups. We performed calculations to determine the expected prevalence of compound heterozygotes for the *FECH* P/LP variant in different ethnic groups. Additionally, we calculated the prevalence of compound heterozygotes for the low-expression SNP locus c.315-48T>C and the P/LP variant in various ethnic groups. We then combined the two sets of data to estimate the total prevalence of EPP in different ethnic groups, as shown in Table 3. Our findings suggested that the distribution of the *FECH* low-expression SNP locus c.315-48T>C in the population significantly influences the population prevalence of EPP. The Chinese population had the second highest gene frequency of this locus among the 10 ethnic groups, which directly contributed to the highest predicted overall prevalence of EPP in the Chinese population among the 10 ethnic groups. This finding underscores the importance of considering the impact of this SNP locus in genetic studies of porphyria. Xiao-Fei Kong and colleagues genotyped 52 Han Chinese volunteers without porphyria and reported that the AF of the *FECH* low-expression SNP locus c.315-48T>C was 41.35% among normal Han Chinese individuals [17]. According to the reference ChinaMAP database, this locus has a gene frequency of 31.79% in the general Chinese population. However, the current literature on EPP in the Chinese population is limited to case reports, family lineage studies, and reports of novel loci. Large-scale epidemiological investigations of EPP in the Chinese population are lacking.

The ChinaMAP database provided a significant number of Chinese population-specific variants, highlighting the genetic traits of porphyria within the Chinese population in comparison to the information in the gnomAD database. Although gnomAD did not include porphyria-associated P/LP variants in Chinese populations or East Asian populations, reports of these variants have been retrieved in East Asian populations such as China, Japan, and Thailand. Additionally, the ChinaMAP database included 23 porphyria-associated P/LP variants. The predicted prevalence of AIP in the Chinese population significantly differed from that in the Japanese population, and the AF of the *FECH* low-expression SNP locus c.315-48T>C in the Chinese population also differed significantly from that in the Japanese population. This finding suggested that using data from the Japanese population as a proxy for data from East Asian populations in some genetics studies lacks rigor, and can sometimes lead to errors in the results.

The prevalence and distribution of porphyria-associated variants differ significantly across ethnic groups. Some mutation sites are found in multiple ethnic populations, while others are unique to specific ethnicities. Some ethnicities have a wide range of mutation sites, while others have very few or none. These differences reflect the significant genetic diversity in porphyria and are associated with higher rates of specific types of porphyria in certain regions and ethnic groups, particularly those affected by founder effects. As a result, these groups have higher carrier and prevalence rates of certain forms of porphyria than other populations.Understanding the genetic characteristics of each type of porphyria in a variety of ethnic populations is crucial for effectively managing patients of different races.

The majority of porphyria genetics studies are retrospective and based on small patient samples, with few large-sample prospective studies using population-based genetic databases. The ChinaMAP database used in this study is a cohort that encompasses various regions and ethnicities in China. This database provides a vast resource for genetic studies in Chinese populations, even in East Asian populations, ensuring the precision and dependability of the experiments. It serves as an exclusive resource and guide for detecting and confirming P/LP variants in genes related to porphyria. We selected ChinaMAP as our source helps to fill in some of the gaps in the study of porphyria genetics in Chinese populations and underscores their unique genetic features. It also assists in exploring the population specificity of porphyria [18]. The ChinaMAP database complements the gnomAD database.

In this study, we estimated the expected carrier rate of the pathogenic AIP variant in the Chinese population to be 1/1059, consistent with the results of Grandchamp B and Hugo Lenglet. The anticipated prevalence of AIP in the Chinese population ranges from 4.72 × 10^−6^ to 9.45 × 10^−6^, with a penetrance ranging from 0.5 to 1%. However, the penetrance of all porphyrias in the Chinese population has not been determined and could not be used as a reference, highlighting the significance of ongoing follow-up and management of porphyria patients.

Our study has several limitations. First, the variants in this study were rated according to the ACMG guidelines. As the guidelines are updated, diagnostic and treatment standards improve, and experimental techniques develop, many of the VUS-P variants identified in this study may be confirmed as P/LP variants in the future. Due to the uncertainty of the pathogenicity of VUS-P variants, we only calculated the carrying rate and prevalence for P/LP variants, and VUS-P variants were not included. However, we have listed some information on VUS-P variants in the ChinaMAP database in Table 5 for reference. Second, the data in ChinaMAP were sourced from natural populations with good metabolism-related traits across China [1], and gnomAD also excluded individuals and their first- and second-degree relatives known to have severe paediatric diseases. Furthermore, we conducted our research under the assumption that ethnic groups adhere to HWE. However, certain groups, such as consanguineous family lines, may not conform to this assumption. As a result, the actual prevalence of porphyria in these specific groups may be greater than what is predicted based on HWE. In summary, our current estimates of the carrier rate and prevalence of porphyria-associated pathogenic mutations should be regarded as “minimal”. Since porphyria has an extremely low penetrance, determining its prevalence in the population by using predicted carrier and prevalence rates necessitates accounting for the penetrance of different types of porphyria. Unfortunately, there are no available data on the penetrance of porphyria in the Chinese population. As a result, the carrier and disease rates for porphyria that we calculated are purely theoretical genetic values. To accurately predict the prevalence in the Chinese population, support from large-scale epidemiological studies is needed.

**Table 5:** Information of VUS-P variants in ChinaMAP

Gene (Type of Porphiria)	DNA change	AA change	DNA Change	HGMD_tag	ACMG Variant	ACMG Evidence of Pathogenicity	REVEL	s-PP3	ChinaMap_Num	ChinaMap_Frq
*ALAD* (ADP)	c.961C>T	p.Pro321Ser	Nonsynonymous SNV		VUS_P	PM2_supporting, PP3	0.657	–	5	0.0002361
	c.922C>T	p.Arg308Cys	Nonsynonymous SNV		VUS_P	PM2_supporting, PP3	0.847	–	3	0.0001417
	c.898G>A	p.Val300Ile	Nonsynonymous SNV		VUS_P	PM2_supporting, PP3	0.463	–	2	0.0000944
	c.866C>A	p.Ala289Asp	Nonsynonymous SNV		VUS_P	PM2_supporting, PP3	0.864	–	2	0.0000944
	c.865G>A	p.Ala289Thr	Nonsynonymous SNV		VUS_P	PM2_supporting, PP3	0.875	–	1	0.0000472
	c.844T>C	p.Phe282Leu	Nonsynonymous SNV		VUS_P	PM2_supporting, PP3	0.876	–	3	0.0001417
	c.804C>A	p.His268Gln	Nonsynonymous SNV		VUS_P	PM2_supporting, PP3	0.712	–	1	0.0000472
	c.802–115A>G	–	Intronic		VUS_P	PM2_supporting, PP3		2 [maxent, spliceAI]	2	0.0000944
	c.787G>A	p.Glu263Lys	Nonsynonymous SNV		VUS_P	PM2_supporting, PP3	0.472	1 [spliceAI]	1	0.0000472
	c.748A>G	p.Met250Val	Nonsynonymous SNV		VUS_P	PM2_supporting, PP3	0.878	–	1	0.0000472
	c.707G>A	p.Arg236Gln	Nonsynonymous SNV		VUS_P	PM2_supporting, PP3	0.85	–	1	0.0000472
	c.692G>A	p.Arg231Gln	Nonsynonymous SNV		VUS_P	PM2_supporting, PP3	0.649	–	38	0.0017945
	c.626G>A	p.Arg209Gln	Nonsynonymous SNV		VUS_P	PM2_supporting, PP3	0.892	5 [ADA, RF, ssplice, maxent, spliceAI]	1	0.0000472
	c.599T>G	p.Phe200Cys	Nonsynonymous SNV		VUS_P	PM2_supporting, PP3	0.888	–	1	0.0000472
	c.523G>A	p.Val175Met	Nonsynonymous SNV		VUS_P	PM2_supporting, PP3	0.677	–	1	0.0000472
	c.500C>T	p.Pro167Leu	Nonsynonymous SNV		VUS_P	PM2_supporting, PP3	0.93	–	2	0.0000944
	c.391C>G	p.His131Asp	Nonsynonymous SNV		VUS_P	PM2_supporting, PP3	0.95	–	1	0.0000472
	c.344T>A	p.Leu115His	Nonsynonymous SNV		VUS_P	PM2_supporting, PP3	0.804	–	2	0.0000944
	c.287C>T	p.Ser96Phe	Nonsynonymous SNV		VUS_P	PM2_supporting, PP3	0.51	–	1	0.0000472
	c.220C>T	p.Arg74Cys	Nonsynonymous SNV		VUS_P	PM2_supporting, PP3	0.4	–	1	0.0000472
	c.100C>T	p.Pro34Ser	Nonsynonymous SNV		VUS_P	PM2_supporting, PP3	0.965	–	1	0.0000472
	c.50G>A	p.Arg17Gln	Nonsynonymous SNV		VUS_P	PM2_supporting, PP3	0.883	–	2	0.0000944
	c.49C>T	p.Arg17Trp	Nonsynonymous SNV		VUS_P	PM2_supporting, PP3	0.887	–	1	0.0000472
	c.28G>A	p.Gly10Ser	Nonsynonymous SNV		VUS_P	PM2_supporting, PP3	0.735	–	1	0.0000472
*HMBS* (AIP)	c.737G>A	p.Arg246His	Nonsynonymous SNV		VUS_P	PP3	0.893	0.5 [spliceAI]	4	0.0001889
	c.34–684G>A	–	Intronic		VUS_P	PM2_supporting, PP3		2 [maxent, spliceAI]	1	0.0000472
	c.34–3C>A	–	Splicing		VUS_P	PM2_supporting, PP3		3 [ADA, RF, spliceAI]	1	0.0000472
	c.65G>A	p.Arg22His	Nonsynonymous SNV		VUS_P	PM2_supporting, PP3	0.859	1 [maxent]	8	0.0003778
	c.211–5G>A	–	Splicing		VUS_P	PM2_supporting, PP3		3 [RF, maxent, spliceAI]	1	0.0000472
	c.330C>G	p.Ile110Met	Nonsynonymous SNV		VUS_P	PM2_supporting, PP3	0.662	–	1	0.0000472
	c.422 + 5G>A	–	Splicing		VUS_P	PM2_supporting, PP3		2 [ADA, RF]	1	0.0000472
	c.471G>T	p.Lys157Asn	Nonsynonymous SNV		VUS_P	PM2_supporting, PP3	0.623	–	3	0.0001417
	c.523C>T	p.Arg175Trp	Nonsynonymous SNV		VUS_P	PM2_supporting, PP3	0.684	–	1	0.0000472
	c.535G>A	p.Glu179Lys	Nonsynonymous SNV		VUS_P	PM2_supporting, PP3	0.723	–	1	0.0000472
	c.569C>T	p.Thr190Ile	Nonsynonymous SNV	DM	VUS_P	PM2_supporting, PP3; PM1; PS4_supporting:The variant was detected in 2 patients [PMID: 11591889, 30556376]	0.555	–	2	0.0000944
	–	–	Intronic		VUS_P	PM2_supporting, PP3		2 [maxent, spliceAI]	1	0.0000472
	c.664G>A	p.Val222Met	Nonsynonymous SNV	DM	VUS_P	PM2_supporting, PP3; PM1; PS4_supporting:The variant was detected in 2 patients [PMID: 9654202]	0.869	–	1	0.0000472
	c.671T>C	p.Val224Ala	Nonsynonymous SNV		VUS_P	PM2_supporting, PP3	0.71	–	5	0.0002361
	c.724G>A	p.Glu242Lys	Nonsynonymous SNV		VUS_P	PM2_supporting, PP3	0.659	–	3	0.0001417
	c.724G>C	p.Glu242Gln	Nonsynonymous SNV		VUS_P	PM2_supporting, PP3	0.585	0.5 [spliceAI]	3	0.0000472
	c.745G>A	p.Ala249Thr	Nonsynonymous SNV		VUS_P	PM2_supporting, PP3	0.932	–	2	0.0000944
	c.787G>T	p.Val263Leu	Nonsynonymous SNV		VUS_P	PM2_supporting, PP3	0.936	–	1	0.0000472
	c.802C>T	p.His268Tyr	Nonsynonymous SNV		VUS_P	PM2_supporting, PP3	0.64	–	2	0.0000944
	c.853C>A	p.Leu285Ile	Nonsynonymous SNV		VUS_P	PM2_supporting, PP3	0.654	–	3	0.0001417
	c.858C>G	p.Asp286Glu	Nonsynonymous SNV		VUS_P	PM2_supporting, PP3	0.808	–	1	0.0000472
	c.949G>A	p.Gly317Ser	Nonsynonymous SNV		VUS_P	PM2_supporting, PP3	0.798	–	1	0.0000472
	c.988G>A	p.Ala330Thr	Nonsynonymous SNV		VUS_P	PM2_supporting, PP3	0.555	–	1	0.0000472
	c.1061C>T	p.Ala354Val	Nonsynonymous SNV		VUS_P	PM2_supporting, PP3	0.723	–	1	0.0000472
*UROS* (CEP)	c.761G>A	p.Gly254Asp	Nonsynonymous SNV		VUS_P	PM2_supporting, PP3	0.601	–	1	0.0000472
	c.742C>T	p.Pro248Ser	Nonsynonymous SNV		VUS_P	PM2_supporting, PP3	0.64	–	1	0.0000472
	c.740C>T	p.Thr247Met	Nonsynonymous SNV		VUS_P	PM2_supporting, PP3	0.724	–	176	0.0083113
	c.713delC	p.Pro238Leufs*2	Frameshift deletion		VUS_P	PVS1_strong, PM2_supporting		–	1	0.0000472
	9c.692C>T	p.Ala231Val	Nonsynonymous SNV		VUS_P	PM2_supporting, PP3	0.662	–	1	0.0000472
	c.606_608delp.Lys202del		Nonframeshift deletion		VUS_P	PM2_supporting, PM4		1 [spliceAI]	1	
	c.565G>T	p.Val189Phe	Nonsynonymous SNV		VUS_P	PM2_supporting, PP3	0.514	–	1	0.0000472
	c.554C>G	p.Ser185Cys	Nonsynonymous SNV		VUS_P	PM2_supporting, PP3	0.41	–	1	0.0000472
	c.530A>C	p.Gln177Pro	Nonsynonymous SNV		VUS_P	PM2_supporting, PP3	0.442	–	1	0.0000472
	c.394+2144G>A	–	Intronic		VUS_P	PM2_supporting, PP3		2 [maxent, spliceAI]	1	0.0000472
	c.394 + 403A>T	–	Intronic		VUS_P	PM2_supporting, PP3		2 [maxent, spliceAI]	2	0.0000944
	c.347G>A	p.Gly116Glu	Nonsynonymous SNV		VUS_P	PM2_supporting, PP3	0.729	0.5 [spliceAI]	1	0.0000472
	c.320T>G	p.Val107Gly	Nonsynonymous SNV		VUS_P	PM2_supporting, PP3	0.931	1 [spliceAI]	1	0.0000472
	c.217T>G	p.Cys73Gly	Nonsynonymous SNV		VUS_P	PM2_supporting, PP3	0.777	1 [spliceAI]	1	0.0000472
	c.169G>A	p.Gly57Arg	Nonsynonymous SNV	FP	VUS_P	PM2_supporting, PP3	0.557	–	4	0.0001889
	c.-27+1113G>A	–	Intronic		VUS_P	PM2_supporting, PP3		2 [maxent, spliceAI]	4	0.0001889
	c.-27+5G>T	–	Intronic		VUS_P	PM2_supporting, PP3		3 [ADA, RF, spliceAI]	4	0.0001897
*UROD* (PCT&HEP)	c.41T>G	p.Leu14Arg	Nonsynonymous SNV		VUS_P	PM2_supporting, PP3	0.952	–	1	0.0000472
	c.74G>A	p.Gly25Glu	Nonsynonymous SNV	DM	VUS_P	PM2_supporting, PP3	0.886	–	1	0.0000472
	c.92C>G	p.Thr31Ser	Nonsynonymous SNV		VUS_P	PM2_supporting, PP3	0.152	–	1	0.0000472
	c.148C>G	p.Arg50Gly	Nonsynonymous SNV		VUS_P	PM2_supporting, PP3	0.3	–	1	0.0000472
	c.299T>A	p.Met100Lys	Nonsynonymous SNV		VUS_P	PM2_supporting, PP3	0.695	–	3	0.0001417
	c.358C>T	p.Arg120Cys	Nonsynonymous SNV	DM	VUS_P	PM2_supporting, PP3	0.376	–	1	0.0000472
	c.364C>T	p.Arg122Trp	Nonsynonymous SNV		VUS_P	PM2_supporting, PP3	0.208	–	6	0.0002833
	c.398A>G	p.Tyr133Cys	Nonsynonymous SNV		VUS_P	PM2_supporting, PP3	0.899	–	1	0.0000472
	c.398A>T	p.Tyr133Phe	Nonsynonymous SNV		VUS_P	PM2_supporting, PP3	0.873	–	1	0.0000472
	c.442C>T	p.Arg148Cys	Nonsynonymous SNV		VUS_P	PM2_supporting, PP3	0.773	–	3	0.0001417
	c.443G>A	p.Arg148His	Nonsynonymous SNV		VUS_P	PM2_supporting, PP3	0.498	–	1	0.0000472
	c.473C>T	p.Pro158Leu	Nonsynonymous SNV		VUS_P	PM2_supporting, PP3	0.971	1 [ADA]	1	0.0000472
	c.474+2_474+3del	–	Splicing		VUS_P	PM2_supporting, PP3		2 [maxent, spliceAI]	1	0.0000472
	c.545A>G	p.Tyr182Cys	Nonsynonymous SNV	DM	VUS_P	PM2_supporting, PP3	0.973	–	1	0.0000472
	c.577C>T	p.Arg193Cys	Nonsynonymous SNV		VUS_P	PM2_supporting, PP3	0.44	0.5 [spliceAI]	4	0.0001889
	c.712C>T	p.Arg238Cys	nonsynonymous SNV		VUS_P	PM2_supporting, PP3	0.222	–	1	0.0000472
	c.719T>C	p.Val240Ala	Nonsynonymous SNV		VUS_P	PM2_supporting, PP3	0.752	–	1	0.0000472
	c.743T>C	p.Leu248Ser	Nonsynonymous SNV		VUS_P	PM2_supporting, PP3	0.751	–	6	0.0002833
	c.745C>T	p.Arg249Trp	Nonsynonymous SNV		VUS_P	PM2_supporting, PP3	0.141	0.5 [spliceAI]	1	0.0000472
	c.758T>A	p.Leu253Gln	Nonsynonymous SNV	DM?	VUS_P	PM2_supporting, PP3	0.512	–	2	0.0000944
	c.773T>C	p.Met258Thr	Nonsynonymous SNV		VUS_P	PM2_supporting, PP3	0.886	2 [ADA, RF]	1	0.0000472
	c.874C>T	p.Arg292Trp	Nonsynonymous SNV		VUS_P	PM2_supporting, PP3	0.868	–	1	0.0000472
	c.919C>G	p.Pro307Ala	Nonsynonymous SNV		VUS_P	PM2_supporting, PP3	0.906	–	3	0.0001417
	c.943G>A	p.Glu315Lys	Nonsynonymous SNV		VUS_P	PM2_supporting, PP3	0.621	2 [ADA, spliceAI]	2	0.0000944
	c.1054G>A	p.Ala352Thr	Nonsynonymous SNV		VUS_P	PM2_supporting, PP3	0.756	–	2	0.0000944
*CPOX* (HCP)	c.1340G>A	p.Arg447His	Nonsynonymous SNV		VUS_P	PM2_supporting, PP3	0.458	–	2	0.0000944
	c.1339C>A	p.Arg447Ser	Nonsynonymous SNV		VUS_P	PM2_supporting, PP3	0.486	–	1	0.0000472
	c.1292A>G	p.His431Arg	Nonsynonymous SNV		VUS_P	PM2_supporting, PP3	0.865	–	65	0.0030695
	c.1257G>A	p.Met419Ile	Nonsynonymous SNV		VUS_P	PM2_supporting, PP3	0.913	0.5 [spliceAI]	2	0.0000944
	c.1256T>C	p.Met419Thr	Nonsynonymous SNV		VUS_P	PM2_supporting, PP3	0.962	0.5 [spliceAI]	2	0.0000944
	c.1205G>C	p.Gly402Ala	Nonsynonymous SNV		VUS_P	PM2_supporting, PP3	0.977	–	1	0.0000472
	c.1115A>G	p.His372Arg	Nonsynonymous SNV		VUS_P	PM2_supporting, PP3	0.78	–	1	0.0000472
	c.1078G>A	p.Ala360Thr	Nonsynonymous SNV		VUS_P	PM2_supporting, PP3	0.629	–	1	0.0000472
	c.1040A>C	p.Lys347Thr	Nonsynonymous SNV		VUS_P	PM2_supporting, PP3	0.718	1 [maxent]	1	0.0000472
	c.1031C>T	p.Ser344Phe	Nonsynonymous SNV		VUS_P	PM2_supporting, PP3	0.468	–	2	0.0000944
	c.1028A>T	p.Asp343Val	Nonsynonymous SNV		VUS_P	PM2_supporting, PP3	0.782	0.5 [spliceAI]	13	0.0006139
	c.980A>G	p.His327Arg	Nonsynonymous SNV	DM	VUS_P	PM2_supporting, PP3, PM3_supporting:Heterozygote of the mutation was detected [PMID: 21103937]	0.974	–	1	0.0000472
	c.953+3A>G	–	Splicing		VUS_P	PM2_supporting, PP3		2 [ADA, RF]	2	0.0000944
	c.928G>C	p.Asp310His	Nonsynonymous SNV		VUS_P	PM2_supporting, PP3	0.248	–	1	0.0000472
	c.891C>G	p.His297Gln	Nonsynonymous SNV		VUS_P	PM2_supporting, PP3	0.897	1 [spliceAI]	1	0.0000472
	c.851C>T	p.Thr284Ile	Nonsynonymous SNV		VUS_P	PM2_supporting, PP3	0.953	–	1	0.0000472
	c.803A>C	p.Glu268Ala	Nonsynonymous SNV		VUS_P	PM2_supporting, PP3	0.703	–	1	0.0000472
	c.767C>G	p.Thr256Ser	Nonsynonymous SNV		VUS_P	PM2_supporting, PP3	0.901	–	2	0.0000944
	c.759T>G	p.His253Gln	Nonsynonymous SNV		VUS_P	PM2_supporting, PP3	0.815	–	1	0.0000472
	c.727G>A	p.Val243Met	Nonsynonymous SNV		VUS_P	PM2_supporting, PP3	0.86	–	2	0.0000944
	c.725G>A	p.Gly242Asp	Nonsynonymous SNV		VUS_P	PM2_supporting, PP3	0.977	–	2	0.0000944
	c.628G>C	p.Val210Leu	Nonsynonymous SNV		VUS_P	PM2_supporting, PP3	0.859	–	1	0.0000472
	c.590G>A	p.Gly197Glu	Nonsynonymous SNV		VUS_P	PM2_supporting, PP3	0.946	–	2	0.0000944
	c.514G>T	p.Gly172Trp	Nonsynonymous SNV		VUS_P	PM2_supporting, PP3	0.773	–	1	0.0000472
	c.434C>T	p.Pro145Leu	Nonsynonymous SNV		VUS_P	PM2_supporting, PP3	0.34	–	1	0.0000472
	c.395C>T	p.Ala132Val	Nonsynonymous SNV		VUS_P	PM2_supporting, PP3	0.471	–	1	0.0000472
	c.284A>C	p.His95Pro	Nonsynonymous SNV		VUS_P	PM2_supporting, PP3	0.736	–	2	0.0000944
	c.178C>G	p.Arg60Gly	Nonsynonymous SNV		VUS_P	PM2_supporting, PP3	0.572	–	2	0.0000963
	c.73C>T	p.Arg25Cys	Nonsynonymous SNV		VUS_P	PM2_supporting, PP3	0.367	–	1	0.0000473
*PPOX* (VP)	c.-176G>A	–	UTR5	DM	VUS_P	PM2_supporting; PS3: This mutation may disrupts splicing [PMID: 27667166]		3 [ADA, RF, spliceAI]	1	0.0000472
	c.-9 + 62G>A	–	Splicing		VUS_P	PM2_supporting, PP3		3 [ADA, RF, spliceAI]	1	0.0000472
	c.78C>G	p.Cys26Trp	Nonsynonymous SNV		VUS_P	PM2_supporting, PP3	0.404	–	1	0.0000472
	c.88-36A>C	–	Intronic		VUS_P	PM2_supporting, PP3		2 [maxent, spliceAI]	4	0.0001889
	c.166C>T	p.Leu56Phe	Nonsynonymous SNV		VUS_P	PM2_supporting, PP3	0.585	–	1	0.0000472
	c.203G>A	p.Gly68Glu	Nonsynonymous SNV		VUS_P	PM2_supporting, PP3	0.932	–	1	0.0000472
	c.236G>A	p.Gly79Asp	Nonsynonymous SNV		VUS_P	PM2_supporting, PP3	0.825	–	1	0.0000472
	c.266G>T	p.Gly89Val	Nonsynonymous SNV		VUS_P	PM2_supporting, PP3	0.594	–	1	0.0000472
	c.269A>T	p.Asp90Val	Nonsynonymous SNV		VUS_P	PM2_supporting, PP3	0.512	–	1	0.0000472
	c.323T>A	p.Leu108Gln	Nonsynonymous SNV		VUS_P	PM2_supporting, PP3	0.845	–	2	0.0000944
	c.339-41A>G	–	Intronic		VUS_P	PM2_supporting, PP3		2 [maxent, spliceAI]	29	0.0013695
	c.350G>A	p.Arg117His	Nonsynonymous SNV	DM	VUS_P	PM2_supporting, PP3	0.738	–	3	0.0001417
	c.361C>A	p.Pro121Thr	Nonsynonymous SNV		VUS_P	PM2_supporting, PP3	0.836	1 [spliceAI]	2	0.0000944
	c.361C>T	p.Pro121Ser	Nonsynonymous SNV		VUS_P	PM2_supporting, PP3	0.809	0.5 [spliceAI]	4	0.0001889
	c.455G>A	p.Arg152His	Nonsynonymous SNV		VUS_P	PM2_supporting, PP3	0.853	–	1	0.0000472
	c.476C>T	p.Ala159Val	Nonsynonymous SNV		VUS_P	PM2_supporting, PP3	0.747	–	1	0.0000472
	c.584G>A	p.Arg195His	Nonsynonymous SNV		VUS_P	PM2_supporting, PP3	0.285	–	2	0.0000944
	c.611G>A	p.Gly204Glu	Nonsynonymous SNV		VUS_P	PM2_supporting, PP3	0.658	1 [spliceAI]	6	0.0002833
	c.668G>A	p.Arg223His	Nonsynonymous SNV		VUS_P	PM2_supporting, PP3	0.073	–	1	0.0000472
	c.688C>G	p.Arg230Gly	Nonsynonymous SNV		VUS_P	PM2_supporting, PP3	0.125	–	17	0.0008028
	c.689G>A	p.Arg230His	Nonsynonymous SNV		VUS_P	PM2_supporting, PP3	0.126	–	1	0.0000472
	c.694G>T	p.Gly232Cys	Nonsynonymous SNV		VUS_P	PM2_supporting, PP3	0.36	–	1	0.0000472
	c.767C>G	p.Pro256Arg	Nonsynonymous SNV	FP	VUS_P	PM2_supporting, PP3	0.032	–	1	0.0000472
	c.808G>C	p.Val270Leu	Nonsynonymous SNV		VUS_P	PM2_supporting, PP3	0.497	3 [ADA, RF, spliceAI]	1	0.0000472
	c.988-11_988–9del	–	Splicing		VUS_P	PM2_supporting, PP3		2 [maxent, spliceAI]	12	0.0005667
	c.1082C>T	p.Pro361Leu	Nonsynonymous SNV		VUS_P	PM2_supporting, PP3	0.268	–	1	0.0000472
	c.1111G>A	p.Gly371Ser	Nonsynonymous SNV		VUS_P	PM2_supporting, PP3	0.878	–	4	0.0001889
	c.1112G>A	p.Gly371Asp	Nonsynonymous SNV		VUS_P	PM2_supporting, PP3	0.571	–	2	0.0000944
	c.1258C>G	p.Pro420Ala	Nonsynonymous SNV		VUS_P	PM2_supporting, PP3	0.247	–	1	0.0000472
	c.1268C>T	p.Thr423Ile	Nonsynonymous SNV		VUS_P	PM2_supporting, PP3	0.295	–	1	0.0000472
	c.1271T>C	p.Leu424Pro	Nonsynonymous SNV		VUS_P	PM2_supporting, PP3	0.722	0.5 [spliceAI]	1	0.0000472
	c.1291+1G>C	–	Canonical splicing	DM	VUS_P	PVS1_strong, PM2_supporting,	0.873	5 [ADA, RF, ssplice, maxent, spliceAI]	1	0.0000472
	c.1391G>A	p.Arg464His	Nonsynonymous SNV		VUS_P	PM2_supporting, PP3	0.159	–	1	0.0000472
*FECH* (EPP)	c.1127T>C	p.Leu376Ser	Nonsynonymous SNV		VUS_P	PM2_supporting, PP3	0.848	–	1	0.0000472
	c.974G>C	p.Arg325Thr	Nonsynonymous SNV		VUS_P	PM2_supporting, PP3	0.565	–	1	0.0000472
	c.864G>A	p.Met288Ile	Nonsynonymous SNV		VUS_P	PM2_supporting, PP3	0.884	–	1	0.0000472
	c.804+6T>A	–	Splicing		VUS_P	PM2_supporting, PP3		3 [ADA, RF, spliceAI]	1	0.0000472
	c.656T>C	p.Met219Thr	Nonsynonymous SNV		VUS_P	PM2_supporting, PP3	0.914	–	1	0.0000472
	c.478T>C	p.Tyr160His	Nonsynonymous SNV		VUS_P	PM2_supporting, PP3	0.943	–	1	0.0000472
	c.466C>T	p.Pro156Ser	Nonsynonymous SNV		VUS_P	PM2_supporting, PP3	0.807	–	1	0.0000472
	c.389C>T	p.Ser130Phe	Nonsynonymous SNV		VUS_P	PM2_supporting, PP3	0.968	–	1	0.0000472
	c.346A>G	p.Thr116Ala	Nonsynonymous SNV		VUS_P	PM2_supporting, PP3	0.599	–	2	0.0000944
	c.326C>T	p.Pro109Leu	Nonsynonymous SNV		VUS_P	PM2_supporting, PP3	0.658	–	1	0.0000472
	c.289G>C	p.Asp97His	Nonsynonymous SNV		VUS_P	PM2_supporting, PP3	0.91	–	1	0.0000472
	c.38G>C	p.Arg13Pro	Nonsynonymous SNV		VUS_P	PM2_supporting, PP3	0.532	–	1	0.0000475
	c.1211C>G	p.Pro404Arg	Nonsynonymous SNV		VUS_P	PM2_supporting, PP3	0.866	–	1	0.0000472
	c.315-48T>C	–	Synonymous SNV	DFP	VUS_P	PS3: The mutation disrupts mRNA splicing [PMID: 11753383]; BA1		0.5 [spliceAI]	6732	0.3179070
	c.935G>T	p.Gly312Val	Nonsynonymous SNV		VUS_P	PM2_supporting, PP3	0.783	1 [maxent]	5	0.0002361
	c.1210C>G	p.Pro404Ala	Nonsynonymous SNV		VUS_P	PM2_supporting, PP3	0.75	–	1	0.0000472
	c.974G>A	p.Arg325Lys	Nonsynonymous SNV		VUS_P	PM2_supporting, PP3	0.46	–	1	0.0000472
	c.200C>T	p.Pro67Leu	Nonsynonymous SNV		VUS_P	PM2_supporting, PP3	0.523	–	1	0.0000472
	c.797C>T	p.Pro266Leu	Nonsynonymous SNV		VUS_P	PM2_supporting, PP3	0.969	–	2	0.0000944
	c.496G>A	p.Val166Ile	Nonsynonymous SNV		VUS_P	PM2_supporting, PP3	0.662	1 [maxent]	1	0.0000472
	c.491G>A	p.Arg164Gln	Nonsynonymous SNV		VUS_P	PM2_supporting, PP3	0.956	2 [maxent, spliceAI]	1	0.0000472
	c.1211C>T	p.Pro404Leu	Nonsynonymous SNV		VUS_P	PM2_supporting, PP3	0.854	–	10	0.0004722
	c.949G>A	p.Glu317Lys	Nonsynonymous SNV		VUS_P	PM2_supporting, PP3	0.665	–	2	0.0000944
	c.370C>T	p.Arg124Cys	Nonsynonymous SNV		VUS_P	PM2_supporting, PP3	0.773	–	1	0.0000472
	c.912G>A	p.Lys304 =	Synonymous SNV		VUS_P	PM2_supporting, PP3		3 [ADA, RF, ssplice]	3	0.0001417
	c.793C>G	p.Leu265Val	Nonsynonymous SNV		VUS_P	PM2_supporting, PP3	0.826	1 [maxent]	1	0.0000472
	c.344G>A	p.Arg115Gln	Nonsynonymous SNV		VUS_P	PM2_supporting, PP3	0.987	–	2	0.0000944
	c.259G>A	p.Asp87Asn	Nonsynonymous SNV		VUS_P	PM2_supporting, PP3	0.583	–	1	0.0000472
	c.2T>C	p.Met1?	Startloss		VUS_P	PM2_supporting; PVS1_supporting	0.552	–	13	0.0006185
	c.643C>T	p.Arg215Trp	Nonsynonymous SNV	DM	VUS_P	PM3: Conpound heterozygous variation with another suspected pathogenic variant p.W301Afs*22 was detected [PMID: 23364466]; PM2_supporting	0.538	–	1	0.0000472
	c.827A>G	p.Tyr276Cys	Nonsynonymous SNV		VUS_P	PM2_supporting, PP3	0.974	–	1	0.0000472

## Data Availability

The datasets supporting the conclusions of this article are available in the ChinaMAP mBiobank repository: http://www.mbiobank.com, and gnomAD Genome V3.0 repository: https://gnomad.broadinstitute.org/blog/2019-10-gnomad-v3-0/.

## Abbreviations

AF: Allele frequencies
P: Pathogenic
LP: Likely pathogenic
VUS-P: Uncertain significance
AD: Autosomal dominant
AR: Autosomal recessive (AR)
HWE: Hardy–Weinberg equilibrium
XLP: X-linked protoporphyria
AIP: Acute intermittent porphyria
HCP: Hereditary coproporphyria
VP: Variegate porphyria
ADP: δ-Aminolevulinic acid dehydratase porphyria
CEP: Congenital erythropoietic porphyria
EPP: Erythropoietic protoporphyria
PCT: Porphyria cutanea tarda
HEP: Hepatoerythropoietic porphyria
ChinaMAP: The China Metabolic Analysis Project
WGS: Whole Genome Sequencing
EAS: East Asian
ASJ: Ashkenazi
AMR: Mixed American
AFR: African/African American
AMI: Amish
FIN: Finnish
NFE: Non-Finnish European
SAS: South Asian
OTH: Other
ACMG: American College of Medical Genetics and Genomics
HGVS: Human Genome Variation Society
EVS: Exome Variant Server
gnomAD: Genome Aggregation Database
REVEL: Rare Exome Variant Ensemble Learner
SVI: Sequence Variant Interpretation
ClinGen: Clinical Genome Resource
SNP: Single nucleotide polymorphism
